# Arginine metabolism and nitric oxide turnover in the ZSF1 animal model for heart failure with preserved ejection fraction

**DOI:** 10.1038/s41598-021-00216-7

**Published:** 2021-10-19

**Authors:** Petra Büttner, Sarah Werner, Svetlana Baskal, Dimitrios Tsikas, Volker Adams, Philipp Lurz, Christian Besler, Sarah Knauth, Martin Bahls, Edzard Schwedhelm, Holger Thiele

**Affiliations:** 1 Department of Cardiology, Heart Center Leipzig at University Leipzig, Strümpellstr. 39, 04289 Leipzig, Germany; 2 Core Unit Proteomics, Hannover Medical School, Institute of Toxicology, Hannover, Germany; 3 Department of Cardiology, University Medicine TU Dresden, Dresden, Germany; 4 Department of Internal Medicine B, University Medicine Greifswald, Greifswald, Germany; 5 DZHK (German Centre for Cardiovascular Research), Partner Site Greifswald, Greifswald, Germany; 6 Institute of Clinical Pharmacology and Toxicology, University Medical Center Hamburg-Eppendorf, Hamburg, Germany; 7 DZHK (German Centre for Cardiovascular Research), Partner Site Hamburg/Kiel/Lübeck, Hamburg, Germany

**Keywords:** Physiology, Biomarkers, Cardiology, Diseases, Medical research, Molecular medicine, Risk factors

## Abstract

Endothelial dysfunction and altered nitric oxide (NO) metabolism are considered causal factors in heart failure with preserved ejection fraction (HFpEF). NO synthase activity depends on the availability of arginine and its derivatives. Thus, we analyzed arginine, associated metabolites, arginine-metabolizing enzymes and NO turnover in 20-week-old female healthy lean (L-ZSF1) and obese ZSF1 rats (O-ZSF1) with HFpEF. Serum, urine and lysates of liver, kidney and heart were analyzed. There were significantly lower lysine (− 28%), arginine (− 31%), homoarginine (− 72%) and nitrite (− 32%) levels in serum of O-ZSF1 rats. Ornithine (+ 60%) and citrulline (+ 20%) levels were higher. Similar results were found in the heart. Expression of arginine consuming enzymes in liver and kidney was unchanged. Instead, we observed a 5.8-fold higher arginase 1 expression, presumably of granulocyte origin, in serum and > fourfold increased cardiac macrophage invasion in O-ZSF1. We conclude that inflammatory cells in blood and heart consume arginine and probably homoarginine via arginase 1 and inducible NO synthase and release ornithine and citrulline. In combination with evidence for decreased NO turnover in O-ZSF1 rats, we assume lower arginine bioavailability to endothelial NO synthase.

## Introduction

Heart failure with preserved ejection fraction (HFpEF) is associated with high morbidity and mortality^1^. While heart failure with reduced ejection fraction (HFrEF) is characterised by the inability of the myocardium to contract and eject properly, systolic function in HFpEF is preserved but a diastolic dysfunction is present. Both, HFpEF and HFrEF patients usually suffer from dyspnoea and exercise intolerance. Myocardial remodelling in HFpEF is characterized by increased vascular and left ventricular (LV) stiffness with impaired relaxation and endothelial dysfunction^1^.

There are different therapeutic treatments accessible for patients with HFrEF, yet these treatments are not effective in HFpEF. So far there are no evidence-based therapies for HFpEF available^2^. An explanation for this is that underpinning pathomechanisms of HFpEF differ from those in HFrEF. Indeed, LV arterial stiffness and endothelial dysfunction may be important for HFpEF pathogenesis^3^. Both conditions are associated with an imbalance of nitric oxide (NO) metabolism. NO synthases (NOS) are sensitive to the availability of the substrates L-arginine (Arg) and L-homoarginine (hArg) and inhibitors, notably asymmetric dimethylarginine (ADMA)^4^. ADMA was found to be associated with worse outcome in cardiovascular syndromes with suspected NO imbalance, and in HFrEF low hArg concentrations were found to be independently associated with mortality^5^. In general, high ADMA blood concentrations are associated with cardio-vascular morbidity and mortality in hypertension, coronary artery disease, and peripheral arterial disease^6^. Low Arg and high ADMA blood concentrations were found to be associated with typical pathophysiological alterations of the heart in HFpEF, e.g., left atrial volume index and average e’^3^.

As human tissue is limited, the underlying mechanisms of the observed imbalance in the Arg/NO pathway are elusive. This obstacle can be resolved by using animal models. Several HFpEF animal models are currently discussed, yet only few fulfill all required criteria of this disease^7^. Obese ZSF1 (O-ZSF1) rats, a F1 hybrid cross breed from male spontaneous hypertensive rats and female Zucker diabetes rats, spontaneously develop hypertension, hyperlipidaemia, glucose intolerance, and exercise intolerance^8^ resulting in a HFpEF phenotype. Hence, these animals offer the possibility to study underpinning pathomechanisms of HFpEF.

In this study, we measured metabolites of the Arg/NO pathway in blood serum and urine of O-ZSF1 rats using previously validated liquid chromatography-tandem mass spectrometry (LC–MS/MS) and gas chromatography-mass spectrometry (GC–MS) approaches. We also determined the gene- and protein-expression of key enzymes of Arg metabolism in several organs using quantitative Realtime-polymerase chain reaction (PCR) and Western Blot analysis. Lean ZSF1 (L-ZSF1) rats served as control group as they do not develop cardiovascular risk conditions and thus no HFpEF phenotype.

## Materials and methods

### HFpEF animal model

All experiments and procedures were performed in accordance with ARRIVE guidelines and relevant animal welfare guidelines and regulations and were approved by the local Animal Research Council, University of Leipzig and the Landesbehörde Sachsen (TVV 30/18).

ZSF1 hybrid rats crossed between a Zucker diabetes fatty female and a spontaneous hypertensive heart failure male rat were used as animal model (ZSF1-Lepr^fa^ Lepr^cp^/Crl, Charles River, Indianapolis, USA). Female O-ZSF1 rats (n = 12) rats at 20 weeks of age were compared with female L-ZSF1 rats (n = 12). All animals were kept at identical conditions under a 12:12 h light/dark cycle with food and water provided ad libitum. Standard chow rich in energy and protein content was delivered by Ssniff (Soest, Germany). Body weight and food intake were recorded every week. Non-invasive echocardiography (Vivid-J, GE Healthcare, Chicago, USA) was used to confirm the presence of HFpEF. For final testing deep anaesthesia was achieved by intraperitoneal injection of 5 mg/kg Xylazin hydrochloride, 100 mg/kg ketamine hydrochloride and 0.1 mg/kg atropine sulfate, based on the individual body weight. Animals were sacrificed by exsanguination.

### Sample processing

Heart, kidney and liver were weighted before further processing. A standardized sample collection routine was used with a prioritization of blood and heart. The heart was cut into base and middle section for histological analysis. The remaining tissue was separated into the left ventricle (LV), septum and right ventricle (RV). One kidney was harvested in total, parts of liver, diaphragm, and cerebrum were collected. Samples of colon and small intestine that were located next to caecum were collected. Tibia was prepared and length was measured. All organs and tissues were immediately snap-frozen in liquid N_2_ and stored at − 80 °C until further use. Frozen samples were pulverised and used for RNA and protein analysis. RNA isolation using up to 30 mg of tissue was done with the RNeasy Kit and QIAShredder (Qiagen, Hilden, Germany) according to the manufacturer’s recommendation. RNA concentration was photometrical determined and 250 or 500 ng specimens were reverse transcribed using Omniscript RT kit with poly dT Primer (Qiagen, Hilden, Germany). For protein analysis 10–20 mg of frozen sample were homogenized in RIPA buffer (10 mM Tris–HCl, pH 8.0, 1 mM EDTA, 0.5 mM EGTA, 1% Triton X-100, 0.1% sodium deoxycholate, 0.1% SDS, 140 mM NaCl) containing a protease and a phosphatase inhibitor mix (Serva, Heidelberg, Germany) and sonicated. Protein concentration was determined using the BCA method (bicinchoninic acid assay, Pierce, Bonn, Germany).

### Determination of Arg, Arg metabolites, nitrite, nitrate, NT-proBNP, arginase 1 and glucose

Established and validated protocols for LC–MS/MS were used to assess serum Arg, ADMA, symmetric dimethylarginine (SDMA) and hArg concentrations^9–11^ (see supplementary materials and methods). Briefly, 25 μL of serum were diluted in methanol that contained the stable isotope labeled internal standards. Thereafter, the analytes were converted into their butyl esters. Analyte concentrations were calculated using calibration curves based on four levels in triplicates. Plate wise quality controls were run in two levels by triplicates. A second analysis was done on the samples to assess coefficient of variation and bias of quality control samples, which was below 15% for all analytes.

Nitrite, nitrate, i.e., the major NO metabolites, creatinine and malondialdehyde (MDA) were measured simultaneously in serum and urine samples by GC–MS using stable-isotope labelled analogs as their internal standards^12^. Arg, hArg and other amino acids in urine were measured by GC–MS^13^. Dimethylamine (DMA), the major metabolite of ADMA was measured by GC–MS^14^. The excretion of nitrite and nitrate was corrected for creatinine excretion and is expressed as µM nitrite or nitrate to mM creatinine (µM/mM).

N-terminal pro Brain Natriuretic Peptide (NT-proBNP) was determined using an ELISA assay according to the manufacturer’s recommendations (abx576280, Hölzel Diagnostika, Cologne, Germany). Arginase 1 was determined in serum using an ELISA assay according to the manufacturer’s recommendations (SEB120Ra, Hölzel Diagnostika, Cologne, Germany). Blood glucose from non-fasted animals was determined using Contour XT with single use strips (Ascensia, Basel, Switzerland).

### Quantitative realtime-PCR

Data on gene expression profiles of enzymes processing Arg in rat organs are limited. Therefore, we first characterized the gene expression profiles in liver, kidney, heart, brain, diaphragm, small intestine and colon of three O-ZSF1 and three L-ZSF1 (Supplementary Fig. 1). Gene expression was determined using Takyon NoRox Sybr Mastermix Blue (Eurogentec, Lüttich, Belgium) according to the manufacturer’s recommendations on a BioRad CFX system (BioRad, Hercules, USA). Primers were designed to bind in different exons or to span a splicing site (see supplemental Table 1 for sequences). The annealing temperature was 60 °C. Standard curves were used for calculation of copy numbers and determination of reaction efficiency. Triplicate measurements were done and samples with standard deviation > 0.250 units were excluded from further analysis. Hypoxanthine phosphoribosyltransferase 1 (Hprt1) and TATA box binding protein (Tbp) were tested as housekeeping genes. Hprt1 was more stable and was further used for expression normalization of the target genes.

**Table 1:** Characteristics of O-ZSF1 and L-ZSF1 at 20 weeks of age.

	L-ZSF1	O-ZSF1	*p*-value
Food intake (g/d)	15 ± 1.3	23 ± 1.5	< 0.0001
Body weight (g)	235 ± 9	468 ± 24	< 0.0001
Heart weight (g)	0.93 ± 0.05	1.38 ± 0.07	< 0.0001
Kidney weight (g)	0.95 ± 0.06	1.54 ± 0.11	< 0.0001
Liver weight (g)	7.20 ± 0.64	18.16 ± 1.9	< 0.0001
Tibia length (mm)	37.1 ± 1.0	37 ± 0.5	0.642
NT-proBNP (pg/ml)	895 ± 371	1200 ± 338	0.047
Glucose (mmol/L)	22.4 ± 3.1	31.2 ± 1.3	< 0.0001
LV-EF (%)	55 ± 12	63 ± 15	0.140
E/e ‘	15.0 ± 2.8	21.7 ± 3.6	< 0.001
Heart rate (bpm)	216 ± 18	214 ± 17	0.812
Heart circumference	106 ± 3	111 ± 4	0.014
LV thickness	10 ± 1.5	12 ± 1.4	0.067
Septum thickness	7.7 ± 1.2	8.9 ± 1.2	0.044
RV thickness	3.6 ± 0.5	3.3 ± 0.5	0.160

LV-EF—left ventricular ejection fraction, E/e’—ratio of mitral peak velocity of early filling (E) to early diastolic mitral annular velocity (*E*'), bpm—beats per minute, LV—left ventricle, RV—right ventricle, NT-proBNP—N terminal B natriuretic peptide.

Mean and standard deviation are given. *P*-value was calculated using Mann–Whitney-U-Test. Histological measurements are given in arbitrary units.

### Western blot analyses

Overall, 10–50 μg of proteins were separated on 10% SDS–polyacrylamide gels. Proteins were transferred to a polyvinylidene fluoride membrane and incubated overnight at 4 °C with the primary antibodies (see supplementary Table 2). Membranes were subsequently incubated with a horseradish peroxidase-conjugated secondary antibody and specific bands visualized by enzymatic chemiluminescence (Super Signal West Pico, Thermo Fisher Scientific Inc., Bonn, Germany) and densitometry quantified using a 1D scan software package (Vision-Capt, Vilber Lourmat, Eberhardzell, Germany). We measured the amounts of arginase 1, arginase 2, glycine amidinotransferase (GATM, AGAT), dimethylarginine dimethylaminohydrolase 1 (DDAH1) and alanine-glyoxylate aminotransferase 2 (AGXT2) (supplementary Table 3,—supplementary summary Fig. 2, single raw files—supplementary Figs. 6–16). Protein expression was normalized to glyceraldehyde-3-phosphate dehydrogenase (GAPDH) (kidney, heart) or alpha tubulin (liver).

### Histological characterization of heart and visualization of NO synthesis in sections of the aortic root

Middle sections of the heart were fixed in 4% paraformaldehyde following section. Material was embedded in paraffin and cut into 4-µm sections, which were then stained for macrophages (CD68 antibody / MCA341R, BioRad, Hercules, USA), or granulocytes (HIS48, ab33760, Abcam, Cambridge, Great Britain). A counterstaining with hematoxylin was done to visualize the tissue structure. Positive cells were counted manually in one complete section per animal.

Heart base was covered with Tissue-Tek® O.C.T.™ Compound (Sakura, Staufen, Germany) in cryomolds and immediately snap frozen. Cryosections of 16-µm thickness were prepared and stored frozen until analysis. Sections with a good view on the aortic root were chosen for the analysis. As the signal intensities showed variances between differed setups we processed and analyzed a pair of L-ZSF1 and O-ZSF1 simultaneously using the same reagents and the same image processing. We adapted published protocols for the use of molecular probes for the visualization of NO synthesis in tissue cross sections^15,16^. Sections were dried for 10 min before 5 µM 4-Amino-5-Methylamino-2',7'-Difluorofluorescein (DAF-FM) (D23844, Molecular probes Thermo Fisher Scientific, Germany) in phosphate buffered saline (PBS) was added. Sections were incubated for 1 h at 37 °C in the dark. Then, sections were rinsed twice in PBS, covered in ROTI®Mount FluorCare (Carl-Roth, Germany), and immediately analyzed using a Keyence BZ-X810 microscope (Keyence, Japan). As negative control, sections were pre-incubated with the NOS antagonist *N*^*G*^*-*nitroarginine methyl ester (L-NAME) (200 µM in PBS) for 30 min at 37 °C. Then, DAF-FM mix including L-NAME at the mentioned concentrations was added. Cell nuclei were stained in all sections using HOECHST 33,342, which was added to DAF-FM solution. DAF-FM signal was analyzed in FITC channel at the same signal intensities for every pair. Overviews of the aorta were prepared with 20-fold magnification (see supplementary Fig. 3). Image-J^17^ was used for image analysis. The aorta was completely marked using the polygon selection tool and the measure tool was used to determine the signal intensities. Median signal intensities of every pair was normalized to one and all analyzed samples were then finally compared group-wise using non-parametric, two sided t-test.

### Statistics and visualization

Data are reported as means ± standard deviation. Group-wise comparisons were done using Mann–Whitney U test or Kruskal Wallis test. A false discovery rate correction was applied for serial measurements from one sample. Gene expression data and Western Blot data were screened for values above or below three standard deviation (SD) units in a SD analysis. No value in Western Blot analysis but seven values in gene expression analysis were excluded from further analysis. Figures were prepared using GraphPad Prism 8 (GraphPad Software, San Diego, USA) or GIMP 2.10.14 (open source software, GIMP team, www.gimp.org).

## Results

### HFpEF rats and controls

O-ZSF1 rats had a 50% higher food intake than L-ZSF1. At sacrifice at an age of 20 weeks, O-ZSF1 had significantly higher body weights as well as heavier hearts, livers and kidneys (*p* < 0.0001 for all, respectively), while their tibia lengths were comparable with those of L-ZSF1 (Table 1). We observed increased NT-proBNP (1.3-fold, *p* = 0.047), glucose (1.4-fold, *p* < 0.0001), E/e’ (1.4-fold, *p* < 0.001) and systolic blood pressure (1.2-fold, *p* < 0.001) in O-ZSF1 rats. LV ejection fraction (LV-EF), heart rate and diastolic blood pressure were comparable (Table 1). In histological analyses, we found that the heart circumference (*p* = 0.014), as well as septum thickness (*p* = 0.044) and by trend LV thickness (*p* = 0.067) were about 10% higher in the O-ZSF1 animals, while RV thickness was comparable in both groups.

### Arg derivatives in serum, urine, LV, kidney and liver

Metabolites of Arg-involving pathways were measured in serum, urine, LV, kidney and liver of 20-weeks old female ZSF1 rats (Table 2).

**Table 2:** Arg and Arg-derived amino acids in O-ZSF1 and L-ZSF1 at the age of 20 weeks.

	L-ZSF1	O-ZSF1	*p* value	O-ZSF1 status
Orn blood (µM)	53.6 ± 12.0	85.9 ± 28.7	0.011	↑
Orn/Cit* urine (µM/mM)	6.3 ± 1.8	8.4 ± 3.8	0.588	↔
Orn liver (nmol/mg)	11.5 ± 2.3	6.6 ± 1.9	< 0.001	↓
Orn kidney (nmol/mg)	4.5 ± 1.0	5.4 ± 0.7	0.777	↔
Orn heart (nmol/mg)	0.05 ± 0.1	0.4 ± 0.6	1.00	↔
Lys blood (µM)	581 ± 112	420 ± 109	0.012	↓
Lys urine (µM/mM)	25.8 ± 13.1	24.6 ± 15.4	1.00	↔
Lys liver (nmol/mg)	31.1 ± 6.1	26.0 ± 3.9	0.168	↔
Lys kidney (nmol/mg)	31.6 ± 3.0	33.4 ± 4.6	0.458	↔
Lys heart (nmol/mg)	16.9 ± 3.4	9.4 ± 1.5	< 0.01	↓
Arg blood (µM)	109 ± 24	74.6 ± 29.6	0.034	↓
Arg urine (µM/mM)	7.9 ± 3.5	8.9 ± 5.8	0.604	↔
Arg liver (nmol/mg)	0.6 ± 0.1	0.7 ± 0.2	1.00	↔
Arg kidney (nmol/mg)	18.0 ± 2.7	19.3 ± 2.6	1.00	↔
Arg heart (nmol/mg)	6.5 ± 1.1	4.2 ± 0.9	< 0.01	↓
Cit blood (µM)	78 ± 10	92 ± 8.4	< 0.01	↑
Orn/Cit* urine (µM/mM)	6.3 ± 1.8	8.4 ± 3.8	0.588	↔
Cit liver (nmol/mg)	1.0 ± 0.3	0.8 ± 0.3	1.00	↔
Cit kidney (nmol/mg)	1.5 ± 0.2	1.7 ± 0.2	1.00	↔
Cit heart (nmol/mg)	3.2 ± 0.7	2.6 ± 0.9	1.00	↔
hArg blood (µM)	1.94 ± 0.49	0.544 ± 0.367	< 0.0001	↓
hArg urine (µM/mM)	0.118 ± 0.05	0.071 ± 0.05	0.07	(↓)
hArg liver (pmol/mg)	101 ± 20.4	61.1 ± 15.6	< 0.001	↓
hArg kidney (pmol/mg)	58.7 ± 9.6	50.5 ± 9.3	1.00	↔
hArg heart (pmol/mg)	72.1 ± 19.9	32.6 ± 7.3	< 0.001	↓
SDMA blood (µM)	0.301 ± 0.028	0.308 ± 0.047	1.00	↔
SDMA liver (nmol/mg)	27.3 ± 5.3	29.8 ± 5.4	1.00	↔
SDMA kidney (pmol/mg)	80.8 ± 10.3	84.0 ± 20.5	1.00	↔
SDMA heart (pmol/mg)	9.5 ± 1.5	6.2 ± 1.3	0.015	↓
ADMA blood (µM)	0.728 ± 0.102	0.844 ± 0.118	0.131	↔
ADMA urine (µM/mM)	0.198 ± 0.085	0.269 ± 0.254	0.364	↔
ADMA liver (nmol/mg)	61.2 ± 21.0	48.0 ± 15.6	0.648	↔
ADMA kidney (pmol/mg)	90.8 ± 22.1	80.0 ± 14.9	1.00	↔
ADMA heart (pmol/mg)	4.7 ± 1.6	7.4 ± 5.6	1.00	↔
DMA urine (µM/mM)	78.3 ± 27.2	70.8 ± 25.3	0.491	↔

Mean and standard deviation are given. P-value was calculated using Mann–Whitney-U-Test. *P*-values were corrected for multiple testing. Measurements in urine were normalized to creatinine. In urine Orn and Cit were measured together (*).O-ZSF1 status—Arrows indicate if concentration was higher, lower or even in O-ZSF1 compared to lean. Arrows in brackets indicate a trend *p* < 0.1

In O-ZSF1, serum lysine (Lys), Arg and hArg concentrations were significantly lower (by at least 28%, all *p* < 0.05) compared to those of L-ZSF1 (Supplementary Fig. 4). The serum concentrations of ADMA and SDMA were comparable in both groups. The highest difference (more than threefold) between the groups was observed for serum hArg concentrations. In urine, where all measurements were creatinine-corrected, hArg concentration by trend was lower in O-ZSF1 while all other amino acids including DMA were comparable in both groups.

In kidney homogenates no differences between the experimental groups were observed for any analyzed amino acid. In liver homogenates of O-ZSF1 Ornithine (Orn) and hArg were significantly lower compared to L-ZSF1 (Supplementary Fig. 5) while concentrations of the other amino acids were comparable. In LV homogenates Lys, hArg and SDMA were lower in O-ZSF1. Orn was only detected in LV homogenate of one L-ZSF1 and in LV homogenate of three O-ZSF1. All other analyzed amino acids were unchanged.

### Gene expression of enzymes processing Arg and its derivatives

We prepared a gene expression profile of Arg consuming enzymes in ZSF1 rat organs (Supplementary Fig. 1). Arg1, coding for arginase 1, had the highest gene expression in liver, moderate expression in kidney and weak expression in the other organs. In kidney, we detected the highest gene expression of Arg2 (coding for arginase 2), Gatm, Ddah1, Ddah2, and Agxt2. While Agxt2 was also highly expressed in liver, its gene expression in other organs was low. In heart, we detected only moderate to weak gene expression of all analyzed enzymes.

**Figure 1:**
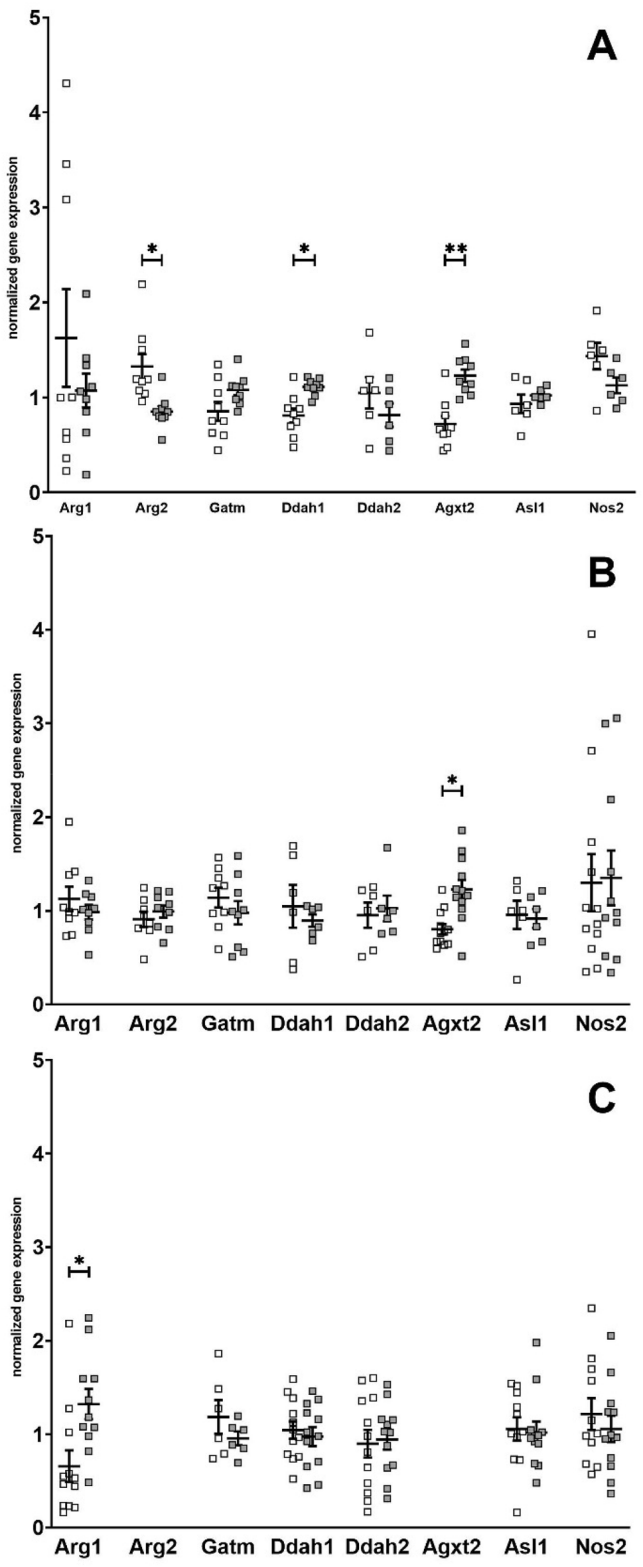
Gene expression of key enzymes involved in arginine metabolism in kidney (**A**), liver (**B**) and heart (**C**) in L-ZSF1 (white squares) and O-ZSF1 (grey squares). Y-axis shows arbitrary units that represent gene expression normalized to Hprt1 and the median of all measurements of the gene. Arg2 and Agxt2 expression in heart was generally low and not detectable in some animals (cycle threshold > 35) irrespective of phenotype and was thus excluded from view. Boxplots visualize the median, 25th and 75th percentiles and minimum/ maximum. *P*-values were calculated using Kruskal–Wallis test and were corrected for multiple testing using the Bonferroni-Dunn method. *p < 0.05, **p < 0.01.

Gene expression was then determined in all samples from liver and kidney (as these organs showed reasonable expression levels) and in heart due to its central role in HFpEF (Fig. 1). In kidney, Ddah1 expression was lower in L-ZSF1 compared to O-ZSF1 (0.81 ± 0.22 vs. 1.11 ± 0.09, *p* = 0.012). Renal Agxt2 expression was also lower in L-ZSF1 compared to O-ZSF1 (0.72 ± 0.25 vs. 1.23 ± 0.2, *p* = 0.001). In contrast, renal Arg2 expression was higher in L-ZSF1 compared to O-ZSF1 (1.33 ± 0.39 vs. 0.85 ± 0.17, *p* = 0.03). In the liver, Agxt2 expression was also lower in L-ZSF1 compared to O-ZSF1 (0.80 ± 0.19 vs. 1.23 ± 0.35, *p* = 0.01). In the heart, Arg1 expression was lower in L-ZSF1 compared to O-ZSF1 (0.66 ± 0.58 vs. 1.32 ± 0.53, *p* = 0.05) (Fig. 1). Argininosuccinate lyase 1 (coded by Asl1) and inducible NO synthase (coded by Nos2) expression was comparable in all tissues in both groups.

### Protein expression of enzymes processing Arg and its metabolites in kidney, liver and heart

We performed Western Blot analysis for all enzymes with differential gene-expression in either liver, kidney or heart and we included GATM due to its relevance in Arg metabolism, i.e., the conversion of Arg to guanidinoacetate or hArg. We first compared the exposure time that was necessary to get a representative signal that was suited to evaluate the protein quantity of each enzyme (see Supplementary Table 3). Only proteins with signals acquired in less than 10 min were evaluated. For normalization of protein content, we determined alpha tubulin and GAPDH (Supplementary Fig. 2). We observed significant regulation of GAPDH in liver (> 50% upregulated in O-ZSF1, *p* < 0.0001) and of alpha tubulin in kidney (> 20% upregulated in O-ZSF1, *p* < 0.05) and heart (> 50% upregulated in O-ZSF1, *p* < 0.0001). Thus, we further normalized the protein expression in liver to alpha tubulin content and in heart as well as kidney to GAPDH.

**Figure 2:**
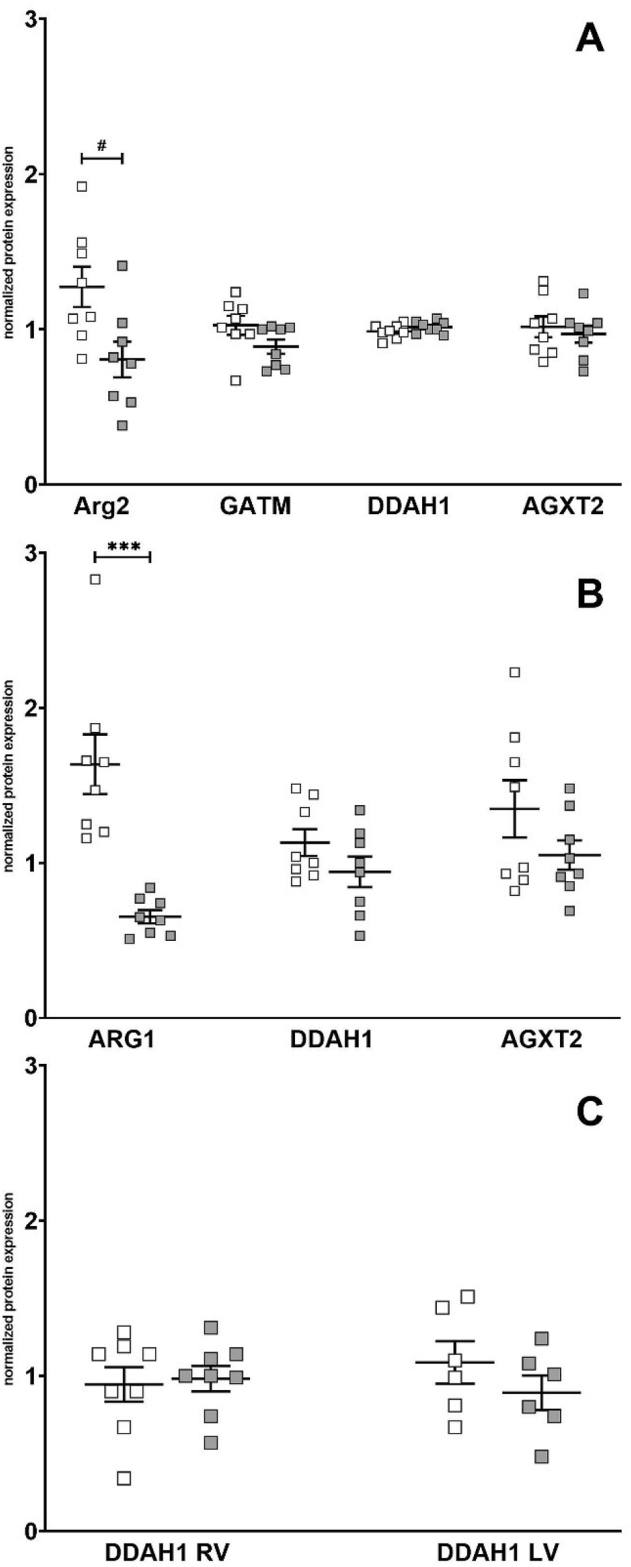
Protein expression of key enzymes involved in arginine metabolism in kidney (**A**), liver (**B**) and heart (**C**) with measurements in right ventricle (RV) and left Ventricle (LV) in L-ZSF1 (white squares) and O-ZSF1 (grey squares). Y-axis shows arbitrary units that represent protein expression normalized to GAPDH (kidney/ heart) or alpha Tubulin (liver). Boxplots visualize the median, 25th and 75th percentiles and minimum/ maximum. *P*-values were calculated using Kruskal–Wallis test and were corrected for multiple testing using the Bonferroni-Dunn method. ^#^*p* < 0.1, ***p < 0.001.

Arginase 2 and GATM were only detected in kidney (Fig. 2). GATM expression was comparable in both experimental groups. Arginase 2 by trend was higher in kidneys of L-ZSF1 compared to O-ZSF1 (1.3 ± 0.37 vs. 0.81 ± 0.33, *p* = 0.07). Arginase 1 was only detected in liver, where it was significantly higher in L-ZSF1 compared to O-ZSF1 (1.64 ± 0.54 vs. 0.65 ± 0.12, *p* < 0.0001) (Fig. 2). DDAH1 was detected in all three organs without group differences. AGTX2 was detected in liver and kidney without group differences, but was undetectable in the heart.

### Arginase 1 concentrations in blood

**Figure 3:**
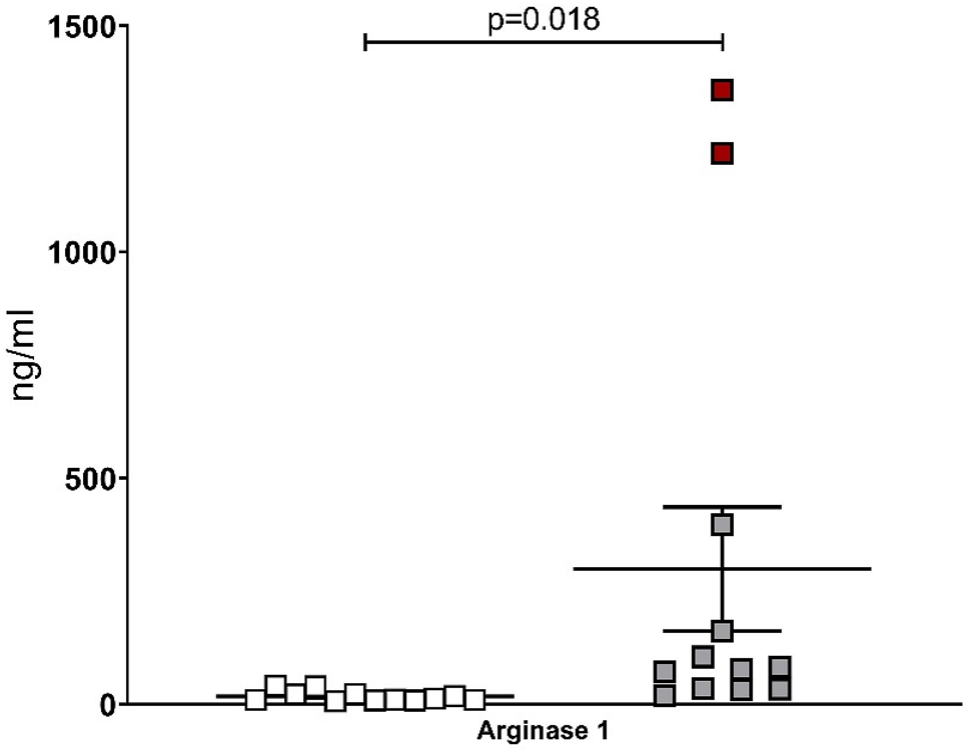
Arginase 1 concentration (ng/ml) in serum of L-ZSF1 (left side) and O-ZSF1 (right side). Boxplots visualize the median, 25th and 75th percentiles and minimum/ maximum. Two samples of the O-ZSF1 group indicated with red boxes were excluded for p-value calculation that was done using Kruskal–Wallis test.

We measured arginase 1 concentration in serum using an ELISA assay. Two O-ZSF1 had arginase 1 concentrations of 1,218 and 1,358 ng/ml, whereas these values differed by more than 10 SD from the mean of the other ten O-ZSF1. When excluding these two animals from further analysis the serum arginase 1 concentrations were still significantly higher in O-ZSF1 (101 ± 112 ng/ml) compared to L-ZSF1 (17.5 ± 11.9 ng/ml, *p* = 0.018) (Fig. 3). Nine O-ZSF1 rats had comparable mean arginase 1 concentrations in the range of 19.5–105 ng/ml; one O-ZSF1 also had a remarkable high arginase 1 concentration of 397 ng/ml.

As serum arginase 1 was the only arginine-consuming enzyme in O-ZSF1 rats that we found to be increased, we analyzed the three animals with exceptionally high serum arginase 1 levels in more detail. We compared them as subgroup G1 with the other nine O-ZSF1 (G2) and with L-ZSF1 to clarify the underlying reason for this observation (Supplementary Table 5). G1 rats were the heaviest O-ZSF1 (494 and 493 g; range in G2 415–485 g, *p* = 0.03). G1 serum also had the highest Orn and Citrulline (Cit) levels and the lowest Lys, Arg and hArg levels (see supplementary Table 5 for group-wise comparison *p*-values). In the LV, Lys, Cit and Arg levels were also significantly lower in G1. Orn was only detected in the LV from G1 rats, while it was absent in G2 rats and only detected once in one L-ZSF1 rat. ADMA and SDMA levels were highest in G1 rats.

We analyzed experimental conditions that may have influenced the three rats: During the experiment G1 rats were not housed in the same cages and had no obvious diseases. At sacrifice, they had no obvious outer or inner abnormalities. They were sacrificed at different days but at comparable day times. Tibia length, heart weight, E/e’ ratio, LV-EF% and NT-proBNP concentrations were comparable to G2 and L-ZSF1.

### Cardiac inflammatory invasion

**Figure 4:**
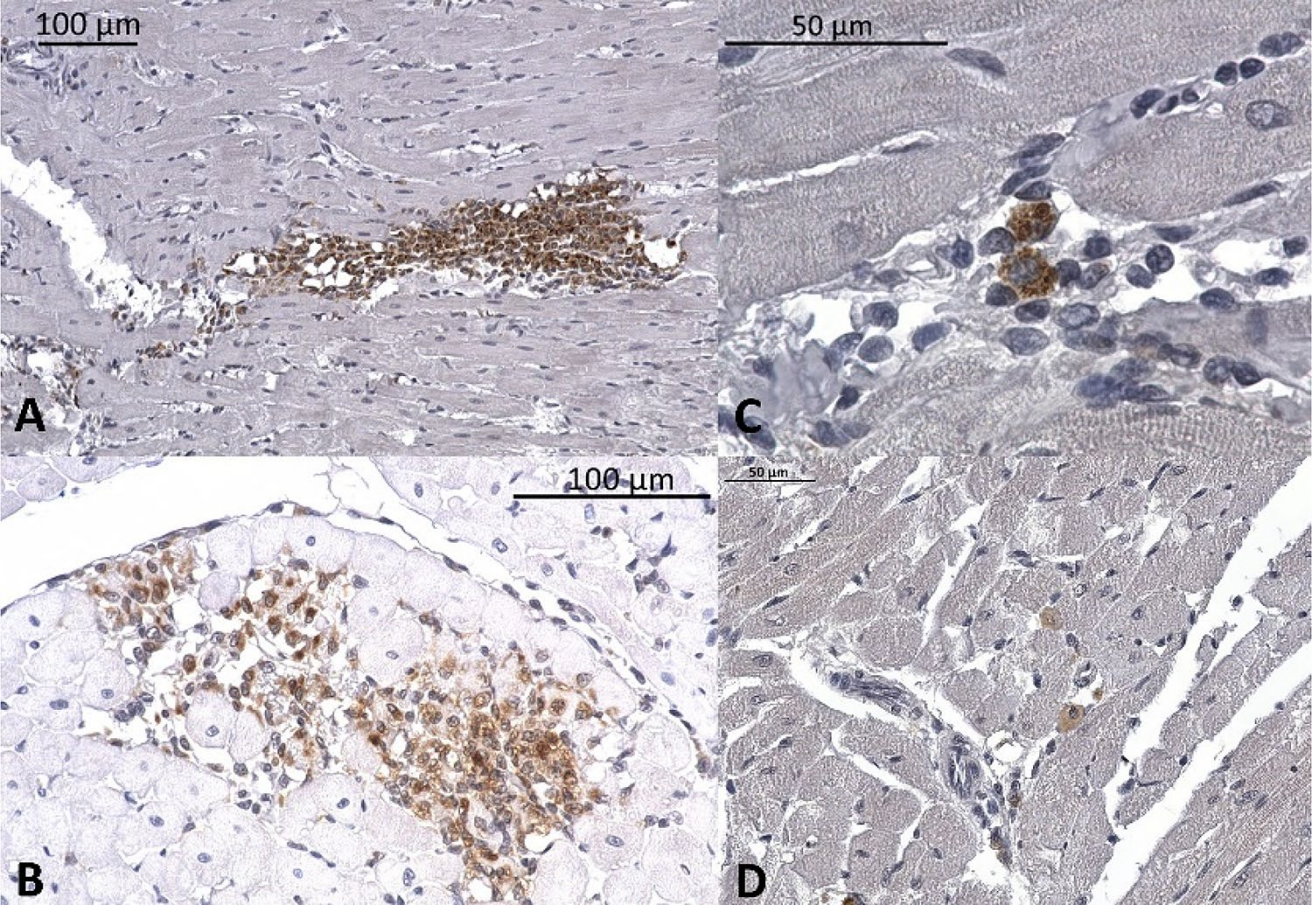
Histological analysis of cardiac macrophage infiltration using CD68 antibody (brown) in sections of O-ZSF1 hearts. Nuclei are stained blue. Exemplary pictures show: (**A**) and (**B**) massive accumulation of macrophages, (**C**) and (**D**) single cells in different magnifications.

We found significantly more macrophages in cardiac sections of O-ZSF1 when compared to L-ZSF1 (49 ± 23 vs. 12 ± 8 cells per section, *p* < 0.001). While macrophages were equally distributed throughout LV, RV and septum in L-ZSF1 we observed pronounced macrophage accumulation in the LV of O-ZSF1 (Fig. 4). Cardiac granulocyte infiltration was rarely observed and there were no differences between both experimental groups.

### NO production in aortic root

NO production was analyzed in cardiac cryosections taken from the heart base including the aortic root directly above the aortic valve (Supplementary Fig. 3). NO production in the aorta of six L-ZSF1 and six O-ZSF1 was detectable and could be blocked using L-NAME. The fluorescence signal was slightly higher (11%) in O-ZSF1 compared to L-ZSF1 (normalized intensity O-ZSF1 1.055 vs. L-ZSF1 0.945, *p* = 0.027).

### Nitrite, nitrate and creatinine in urine

Nitrite and nitrate are major circulating and urinary metabolites of NO. We measured nitrite and nitrate in urine as surrogates of whole body NO synthesis in the rats. Urinary creatinine excretion was lower in O-ZSF1 (0.910 ± 0.386 mM) compared to L-ZSF1 (2.42 ± 1.41 mM; *p* = 0.002). Thus, all measurements in urine were corrected for creatinine concentration. Creatinine-corrected nitrate and nitrite excretion was higher in O-ZSF1 compared to L-ZSF1: nitrate 135.4 ± 38.2 µM/mM vs. 78.8 ± 27.7 µM/mM (*p* = 0.0004), nitrite 11.2 ± 3.1 µM/mM vs. 5.0 ± 2.4 µM/mM, (*p* = 0.00002). Urinary DMA is a surrogate of whole body ADMA synthesis^18^. Creatinine-corrected DMA excretion did not differ between O-ZSF1 and L-ZSF1 (Table 2).

### Nitrite, nitrate, creatinine and MDA in serum

We measured nitrite, nitrate, creatinine and MDA in serum samples of 12 O-ZSF1 and 11 L-ZSF1 rats. The serum nitrite concentrations were lower in the O-ZSF1 (109 ± 18 µM) compared to L-ZSF1 rats (160 ± 51 µM; *p* = 0.004). Serum nitrate concentrations tended to be lower in O-ZSF1 (90.4 ± 3.7 µM) compared to L-ZSF1 rats (93.4 ± 4.4 µM; *p* = 0.090). Creatinine tended to be higher in O-ZSF1 (96.3 ± 32.9 µM) compared to L-ZSF1 (76.4 ± 18.7 µM; *p* = 0.093). MDA concentrations were higher in O-ZSF1 (3.25 ± 0.69 µM) compared to L-ZSF1 (2.43 ± 0.26 µM; *p* = 0.0014).

## Discussion

**Figure 5:**
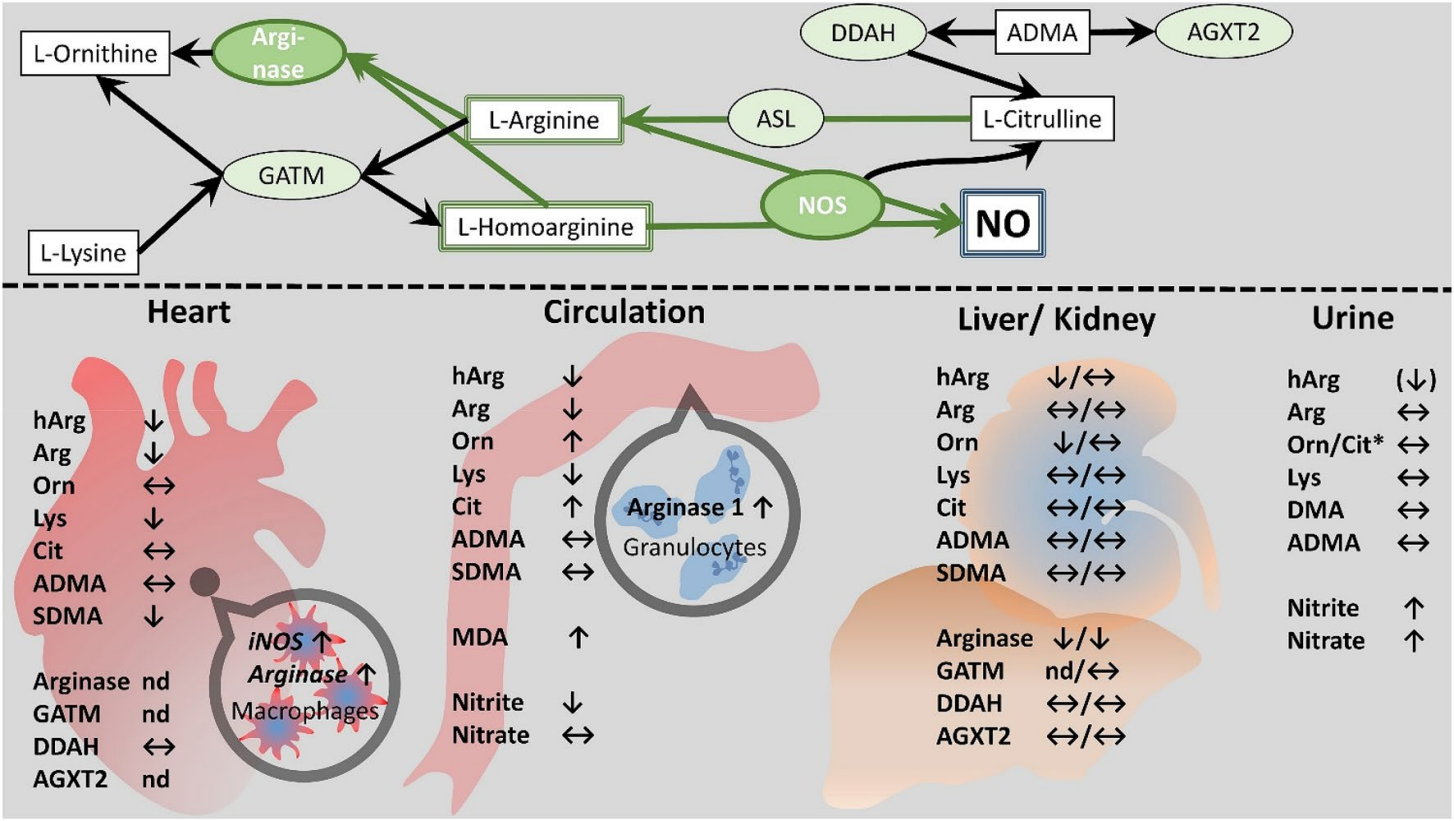
Simplified schematic of findings and proposed status of Arg-involving pathways related to NO in heart, circulation, liver and kidney of O-ZSF1 and L-ZSF1 rats. Top—Enzymes (green) with substrates and metabolites (white). Bottom—Analyzed rat organs and tissues with arrows indicating the changes (*p* < 0.05) compared to L-ZSF1. ↑ higher in O-ZSF1, ↔ no differences, ↓ lower in O-ZSF1. Arrow in brackets indicates a trend (*p* < 0.1). *Orn/Cit were measured together in Urine. iNOS and Arginase were not determined in cardiac macrophages directly—the depicted regulation is hypothesis generating only.

We analyzed metrics of NO turnover as well as arginine metabolism associated amino acids and derivatives in serum, urine, liver, kidney and heart of O-ZSF1 rats—a model for HFpEF—and compared them with healthy L-ZSF1 rats. In O-ZSF1 we found: (1) a pronounced decrease of Arg and hArg in blood and heart; (2) an up-regulation of presumably granulocyte derived arginase 1 accompanied by increased end product Orn in blood; (3) indicators for decreased NO production in serum; (4) a down regulation of arginase 1 and 2 in kidney and liver; and (5) no evidence for increased excretion of hArg and Arg through the kidneys. A simplified overview of arginine metabolizing enzymes and our findings is summarized in Fig. 5.

### Rationale

Different pathomechanisms for HFpEF have been suggested, with systemic, cardiac and cardiomyocyte-specific changes contributing to initiation and progression of HFpEF to different extents at different stages^19^. The impact of the Arg/NO pathway in HFpEF pathophysiology has been demonstrated in a high fat diet mouse model where the animals developed HFpEF characteristics following continuous NOS inhibition by the synthetic L-NAME^4^, a strong yet non-specific synthetic NOS inhibitor. The *N*^G^-methylated Arg derivatives ADMA and SDMA are endogenous NOS activity inhibitors^20^. In HFpEF patients pathophysiological alterations of the heart and individual exercise capacity were found to relate to low Arg (endogenous NOS substrate) and high ADMA (endogenous NOS activity inhibitor) concentrations and a decreased Arg/ADMA ratio in the blood^3^. Higher circulating concentrations of hArg were found to be associated with milder symptoms and improved training effects in HFpEF patients^3,21^. The aim of the present work was to determine the status of Arg-involving enzymes in O-ZSF1, which spontaneously manifest HFpEF over time because of obesity, hypertension and diabetes mellitus^7^. In confirmation of previous studies, O-ZSF1 rats in our study developed obesity, hypertension, diabetes and HFpEF (higher NT-proBNP, diastolic dysfunction, normal LV-EF) at an age of 20 weeks with LV remodeling^8,22^. We analyzed the expression of genes and proteins in Arg/NO-involving pathways and their multiple metabolites in serum, urine and relevant organs of O-ZSF1 and L-ZSF1 as a control. In addition, MDA was determined as a measure of oxidative stress, notably lipid peroxidation^23^.

### Status of serum Arg derivatives and endothelial NO synthesis in the aortic root of O-ZSF1

We found remarkable differences between the experimental groups for all analytes except for ADMA, SDMA and nitrate in serum. While the NOS and arginase substrates Arg and hArg were lower in O-ZSF1, concentrations of their turnover products Orn (indicating increased arginase activity) and Cit (indicating increased NOS activity) were higher. When we analyzed NO synthesis in cryosections of the aortic root we observed comparable and even slightly higher measurements in the HFpEF rats whereas the effect could be blocked using the NOS inhibitor L-NAME. This finding is contrary to recent findings showing lower NO-associated relaxation of aorta^24^ in O-ZSF1. We assume that the reported endothelial dysfunction in the aorta^24^ may have other reasons than impaired NO synthesis. Unfortunately, we collected no other material to determine NO synthesis in other tissue than aortic root. Noteworthy, in HFpEF patients not only general present conduit endothelial dysfunction but especially microvascular endothelial dysfunction of the coronaries is discussed as important pathomechanism^25^. Future studies in ZSF1 rats should thus include peripheral arteries and microvasculature to detect and characterize more detailed the status of endothelial NOS activity, NO availability and endothelial dysfunction in HFpEF.

### Regulation of Arg-metabolizing enzymes in liver and kidney of ZSF1 rats

To explain the imbalance in Arg metabolism in the serum of HFpEF rats we characterized the expression of the metabolizing enzymes in different tissues and organs. Arg is a substrate for arginase 1 and 2 as well as for GATM. In line with human data, Arg1 in rats is mainly expressed in the liver, while Arg2 and Gatm are highly expressed in the kidneys. Both, arginase 1 and arginase 2, were downregulated on a protein expression level in liver and kidney, whereas Arg2 was also transcriptionally downregulated in the kidney. GATM was generally unchanged why we conclude that the enzyme is not responsible for the altered Arg concentration. Arg derivatives and metabolites in kidney lysates were unchanged while in the liver we found a significantly lower concentration of Orn. In this context it is noteworthy that arginase activity is usually proportional to the amount of arginase protein and arginase gene expression^26^. We conclude that the observed lower Arg and hArg level combined with higher Orn levels in blood are not a consequence of increased consumption by arginase in kidney and liver.

### Regulation of arginases in blood and heart and the fate of Arg and hArg of ZSF1 rats

Arginase 1 is typically regarded as a key enzyme in hepatic urea cycle, but it is also expressed in granulocytes^27^, monocytes and macrophages^28^. Unfortunately, we did not collect these cells during the experiment, but we determined the free enzyme in serum. While arginase 1 levels in serum were equally low in L-ZSF1, levels were at least threefold higher in the O-ZSF1 rats. We did not detect arginase 1 in the heart on a protein level but we found Arg1 gene expression to be significantly higher in O-ZSF1. The detected expression may be attributable to the observed increased macrophage infiltration in the heart. Notably, Arg and hArg levels were markedly lower in blood and heart homogenates in the O-ZSF1. Three O-ZSF1 had exceptionally high arginase 1 concentrations and the arginase metabolite Orn was highest in the serum of these animals but was below the detection limit in all other animals except one L-ZSF1. It was reported that inflammatory tissue macrophages express arginase 1 and increase inducible NOS associated NO production leading to Arg depletion in the microenvironment^28,29^. Notably, arginase 1 controls expression of inducible NOS and an imbalance between both enzymes resulted in aggravated inflammation in a mouse model^28^. The expression of Nos2, coding for inducible NOS, was comparable in kidney, liver and heart of both experimental groups in our study. Activity of inducible NOS could not be determined due to sample limitations.

Cardiac macrophage infiltration is triggered by T helper cell cytokines^27^ and it was shown that these cells are also activated in human HFpEF^30^. Typically, the first line of inflammatory infiltration in ischemic heart disease is represented by granulocytes, which are then followed by macrophages and lymphocytes. However, in non-ischemic HF granulocytes are thought to play a minor role compared to monocytes and lymphocytes^31^. To complete our analysis, we also checked the O-ZSF1 hearts for granulocyte infiltration but found no evidence for an increase of those cells.

Importantly, O-ZSF1 develop kidney failure over time^32^ and hArg is excreted via the kidneys. Higher urine levels of hArg correlate with higher estimated glomerular filtration rate as a measure of kidney function; lower urine hArg levels are associated with adverse outcomes in renal disease^33^. In urine of O-ZSF1 excretion rates of most Arg metabolites were comparable, but the excretion rate of hArg was again lower. SDMA is cleared from circulation via the kidneys and is accepted as a sensitive parameter of renal function^6^. As SDMA was comparable in all animals, we conclude that O-ZSF1 rats showed no evidence for progressed kidney failure and lower hArg concentrations are presumably not a consequence of this condition.

### Consumption of Arg and hArg by NOS in O-ZSF1

Arginase 1 and 2 are also expressed in endothelial cells where they are supposed to diminish NO synthesis^26^. Unfortunately, in this study we did not isolate endothelium (except cryopreserved aortic root) from the rats to characterize arginase and NOS expression patterns and their activity in the endothelium. Nevertheless, we can assume the impact of the low concentrations of Arg and hArg on NOS and want to discuss the evidence we found. Although, the affinity of NOS for hArg is much lower than for Arg, a role of hArg in NO signaling has been proposed. Independent associations between low hArg levels and increased mortality in cardiovascular diseases with an involvement of endothelial dysfunction underpin this assumption^5,34,35^. Low hArg concentrations combined with low Arg concentrations as observed in our study may have an impact on endothelial NOS activity. This should be addressed in another study. It was reported that endothelial NOS prefers endogenous Arg derived from intracellular protein turnover or produced by argininosuccinat-lyase (ASL), an enzyme primarily expressed in liver and kidney^26^. Importantly, ASL can also utilize Cit as a substrate^26^, which was found to be higher in the serum of O-ZSF1. We thus analyzed Asl1 gene expression in liver, kidney and heart, but found no differences between the two study groups. ASL protein levels were reported to be closely associated with gene expression changes^36^ and were thus not further analyzed.

### Implications for NO turnover in ZSF1 rats

NO is extremely short lived in living organisms, especially in the circulation, and evades direct measurement. NO is oxidized to nitrite and nitrate which can be measured in fluids and tissues and serve as surrogates for NO^37^. Therefore, we determined the concentration of nitrite and nitrate in serum and urine samples of the rats by a reliable GC–MS method^38^. The lower serum concentration of nitrite in O-ZSF1 is a strong indication of a lower NO bioavailability in the circulation, notably in the vessel endothelium^39^. This is supported by the higher oxidative stress measured as increased MDA in O-ZSF1. MDA is generally considered a biomarker of oxidative stress, notably of lipid peroxidation^23^. Nevertheless, the higher nitrate excretion in O-ZSF1 urine suggest that O-ZSF1 are associated with higher whole-body NO synthesis, presumably due to higher activity of the inducible NOS isoform as discussed above. Importantly, endothelium independent nitrate-nitrite-NO production was discussed in HFpEF as well^40^ and needs to be considered with regard to the massive increase of urinary nitrate as observed in O-ZSF1. Although the renal clearance of nitrate was also increased in O-ZSF1 the differences in blood concentrations were more pronounced. Under normoxic conditions, nitrate-nitrite-derived NO has only minor vasodilatory effects but these are increased in hypoxia. In addition, while NO synthesis by Arg utilizing NOS is inhibited by hypoxia and acidosis, free conversion of nitrite to NO is increased^40^.

Finally, we want to mention that all ZSF1 rats in this study show significantly different nitrite and nitrate concentrations compared to healthy Wistar Kyoto male and female rats^41^. We do not know whether this is a consequence of isolated breeding of the mother and father strains or a consequence of the leptin receptor defect. In this context it should be kept in mind that two third of the L-ZSF1 rats shall be heterozygous for one of the two inherited leptin receptor mutations. Importantly, it was shown in male Zucker diabetic fatty rats that arginine supplementation improved NO turnover in adipose tissue and led to decreased ADMA concentrations^42^.

### Post-translational Arg modifications in O-ZSF1

ADMA and SDMA are Arg metabolites of post-translational modification of Arg dimethylation in proteins and regular proteolysis^26^. We sacrificed the animals at a comparably young age at an early stage of HFpEF and found no differences in ADMA and SDMA. It needs to be analyzed how ADMA and SDMA develop over lifetime, especially older age. Kidney and liver play major roles in direct renal clearance and enzymatic metabolism of ADMA^6^. ADMA is metabolized by AGXT2, which we found to be higher on gene level, but unchanged on a protein level. ADMA is mainly hydrolyzed by DDAH1, with DDAH2 seeming to be of minor importance^6^. Although DDAH1 gene expression was higher in O-ZSF1 kidneys and protein levels of DDAH1 were detectable in kidney, liver and heart, the latter were unchanged. Increased renal clearance of ADMA or its main degradation product DMA is also not supported by our data. We found Cit, the second product of DDAH activity, was higher in the blood of O-ZSF1 but unchanged in kidney, liver and heart lysates. This indicates that DDAH activity in the organs is unchanged and that increased Cit in the circulation is more likely attributable to increased inducible NOS activity. Actually, the picture is incomplete without an analysis of AGXT2 and DDAH1 activity. Unfortunately, currently accessible colorimetric AGXT2^43^ and DDAH activity^44^ assays were insufficient in our hands. We experienced severe interference of the native tissue lysate color, which differed significantly between the experimental groups, with the detection wavelengths. A limitation in remaining sample amounts did not allow for further assay improvement, for instance by sample clean up prior activity measurements. In addition, it is worth mentioning that Cit is a major metabolite of other pathways such as the urea cycle, which we did not study.

### Limitations

Findings in animal models need to be interpreted carefully concerning comparability with humans. The murine metabolism as well as expression profiles of genes and proteins may differ. As animals were in early adolescence at sacrifice, hormone status presumably differed from the typical patient group. We analyzed rats at an age of 20 weeks when HFpEF is already manifest but progression is at an early stage. It is of importance to characterize the alterations in Arg metabolism prospectively.

## Conclusion

Arg derivatives and turnover products as well as nitrite and nitrate concentrations are altered in O-ZSF1. This may mainly be attributable to increased Arg and hArg turnover in inflammatory cells. Consequentially, reduced accessibility of Arg and hArg in the heart and blood vessels could have an impact on endothelial NOS activity. Further, higher oxidative stress as indicated by increased MDA, may worsen the NO imbalance. Normalization of the arginine metabolism by supplementation of Arg or hArg may represent a promising intervention strategy in HFpEF treatment. Further, Arg and hArg levels could be used for monitoring of disease progression or interventional success.

## Supplementary Information

Supplementary Information.

## Data Availability

The datasets generated during and/or analyzed during the current study are available from the corresponding author on reasonable request.
