# Supplementary material for: Arginine metabolism and nitric oxide turnover in the ZSF1 animal model for heart failure with preserved ejection fraction
Source: Sci Rep. 2021 Oct 19;11:20684. doi: 10.1038/s41598-021-00216-7 (PMC8526609; doi:10.1038/s41598-021-00216-7)

Arginine metabolism and nitric oxide turnover in the ZSF1 animal model for heart failure with preserved ejection fraction

*Petra Büttner^1^, Sarah Werner^1^, Svetlana Baskal^2^, Dimitrios Tsikas^2^, Volker Adams^3^, Philipp Lurz^1^, Christian Besler^1^, Sarah Knauth^1^, Martin Bahls^4,5^, ^+^Edzard Schwedhelm^6,7^, ^+^Holger Thiele^1^

^1^ Heart Center Leipzig at University Leipzig, Department of Cardiology, Leipzig, Germany

^2^ Institute of Toxicology, Core Unit Proteomics, Hannover Medical School, Hannover, Germany

^3^ Department of Cardiology, University Medicine TU Dresden, Dresden, Germany

^4^ Department of Internal Medicine B, University Medicine Greifswald, Greifswald, Germany

^5^ DZHK (German Centre for Cardiovascular Research), partner site Greifswald, Greifswald, Germany

^6^ Institute of Clinical Pharmacology and Toxicology, University Medical Center Hamburg-Eppendorf, Hamburg, Germany

^7^ DZHK (German Centre for Cardiovascular Research), partner site Hamburg/Kiel/Lübeck, Hamburg, Germany

**Correspondence:** Petra Büttner; ORCID: 0000-0003-0799-4725

Phone: +49 341 865 252605

Fax: +49 341 8651674

E-Mail: Petra.Buettner@medizin.uni-leipzig.de

Heart Center Leipzig at University of Leipzig; Strümpellstr. 39, 04289 Leipzig, Germany

*These authors contributed equally to this work.

**Supplementary table 1: Primer sequences**

| **Gene** | **full name** | **Forward** | **Reverse** | **Amplicon size (bp)** |
| --- | --- | --- | --- | --- |
| Hprt1 | hypoxanthine phosphoribosyltransferase 1 | CCCAGCGTCGTGATTAGTGA | GGCCTCCCATCTCCTTCATG | 163 |
| Agxt2 | Alanine-Glyoxylate Aminotransferase 2 | CCGCCAGCCTCTTCCTAAAA | CTTCCAGCTTTGTGACACGC | 137 |
| Arg1 | Arginase 1 | CCCGCAGCATTAAGGAAAGC | TGAAAGGGGCTGTCATTGGG | 113 |
| Arg2 | Arginase 2 | GGGCAGCCTCTTTCCTTTCT | GCAGGCTCCACATCTCGTAA | 131 |
| Asl | Argininosuccinate lyase | TCTGTGGAATGTGGACCTGC | CTTGTCCAGGCCTTGCAGTA | 109 |
| Ddah1 | Dimethylarginine dimethylaminohydrolase 1 | TGACAAGCTCACTGTACCGG | ACCTTTGCGCTTTCTGGGTA | 117 |
| Ddah2 | Dimethylarginine dimethylaminohydrolase 2 | CTGACTGATCACCCCTACGC | AACCCAGGACGCAGAAAGAG | 77 |
| GATM | Glycine amidinotransferase | CGCAGAGAAGCCAGGTTACA | GGAGAGCACAAGACCAGGTC | 155 |
| TBP | TATA-box binding protein | ACAGCCTTCCACCTTATGCT | GATTGCTGTACTGAGGCTGC | 238 |

| **Supplementary table 2: Antibodies used for Western Blot analysis.** DDAH1 - Dimethylarginine dimethylaminohydrolase 1, DDAH2 - Dimethylarginine dimethylaminohydrolase 2, GATM - Glycine amidinotransferase | | | | | |
| --- | --- | --- | --- | --- | --- |
| **protein** | **manufacturer** | **order number** | **dilution** | **size** |  |
| GAPDH | Hy Test | 5G4 | 1:30000 | 40kDa |  |
| Arginase-1 | Cell Signaling | 93668 | 1:1000 | 40kDa |  |
| Arginase-2 | Cell Signaling | 55003 | 1:1000 | 40kDa |  |
| GATM | Thermo Fisher | PA5-76957 | 1:1000 | ~45kDa |  |
| DDAH1 | Thermo Fisher | PA5-50610 | 1:1000 | ~35kDa |  |
| AGXT2 | Thermo Fisher | PA5-103587 | 1:1000 | ~55kDa |  |
| aTubulin | Abcam | ab7291 | 1:10000 | 55kDa |  |

**Supplementary table 3:** Exposure time and protein concentrations used for the specific Western Blot detection of key enzymes in arginine metabolism.

|  | **Liver** | | **Kidney** | | **Heart** | |
| --- | --- | --- | --- | --- | --- | --- |
|  | **exposure time** | **protein concentration** | **exposure time** | **protein concentration** | **exposure time** | **protein concentration** |
| **GAPDH** | 13sec | 10µg | 6sec | 10µg | 4sec | 25µg |
| **a Tubulin** | 75sec | 10µg | 13sec | 50µg | 74sec | 25µg |
| **Arg1** | 4sec | 10µg | *>10min* | *50µg* | *>10min* | *50µg* |
| **Arg2** | *>10min* | *50µg* | 90sec | 25µg | *>10min* | *25µg* |
| **GATM** | *>10min* | *50µg* | 15sec | 10µg | *>10min* | *25µg* |
| **DDAH1** | 67sec | 10µg | 7sec | 10µg | 7sec | 25µg |
| **AGXT2** | 2min20sec | 50µg | 2min20sec | 50µg | *>10min* | *50µg* |

**Supplementary table 4:** Concentrations (µmol/L) of arginine derivatives in obese ZSF1 rats with HFpEF (n=12) and lean controls (n=12). Mean ± standard deviation (SD), the concentration change in obese rats compared to lean and the p-value for group-wise comparison with multiple testing correction using false discovery rate are presented. Below the table, correlation between arginine derivatives is described by Pearson coefficient (PC) and the two-sided p-value. Significant correlations are printed in bold. ns – not significant

|  |  | **Ornithine** | **Lysine** | **Arginine** | **Citrulline** | **Homo-arginine** | **SDMA** | **ADMA** |
| --- | --- | --- | --- | --- | --- | --- | --- | --- |
|  | **obese mean ± SD** | 86±29 | 420±109 | 75±30 | 92±8 | 0.544±0.37 | 0.308±0.05 | 0.844±0.12 |
|  | **lean mean ± SD** | 54±12 | 581±112 | 109±24 | 78±10 | 1.942±0.50 | 0.300±0.03 | 0.728±0.10 |
|  | **fold-change** | 1.6 | 0.7 | 0.7 | 1.2 | 0.3 | 1.03 | 1.2 |
|  | **p-value** | 0.011 | 0.012 | 0.033 | 0.008 | <0.0001 | ns | ns |
| **Ornithine** | **PC** |  | -0.360 | **-0.659** | **0.616** | **-0.568** | **0.630** | **0.630** |
|  | **p-value** |  | 0.084 | **4.6E-04** | **0.001** | **0.004** | **0.001** | **0.001** |
| **Lysine** | **PC** | -0.360 |  | **0.783** | -0.333 | **0.737** | 0.182 | 0.102 |
|  | **p-value** | 0.084 |  | **6.0E-06** | 0.112 | **4.0E-05** | 0.395 | 0.635 |
| **Arginine** | **PC** | **-0.659** | **0.783** |  | -0.265 | **0.677** | -0.083 | 0.017 |
|  | **p-value** | **4.6E-04** | **6.0E-06** |  | 0.210 | **2.8E-04** | 0.699 | 0.938 |
| **Citrulline** | **PC** | **0.616** | -0.333 | -0.265 |  | **-0.540** | **0.513** | **0.669** |
|  | **p-value** | **0.001** | 0.112 | 0.210 |  | **0.006** | **0.010** | **3.5E-04** |
| **Homo-arginine** | **PC** | **-0.568** | **0.737** | **0.677** | **-0.540** |  | -0.065 | -0.295 |
|  | **p-value** | **0.004** | **4.0E-05** | **2.8E-04** | **0.006** |  | 0.764 | 0.162 |
| **ADMA** | **PC** | **0.630** | 0.102 | 0.017 | **0.669** | -0.295 | **0.729** |  |
|  | **p-value** | **0.001** | 0.635 | 0.938 | **3.5E-04** | 0.162 | **5.4E-05** |  |

**Supplementary Table 5:** Arginine derivatives in blood, kidney, liver and heart of obese HFpEF rats (G1, G2) and lean controls (G3) with subgrouping of obese HFpEF rats according to highest (G1) and median (G2) serum Arginase 1 concentrations (see first line in table). Orn – Ornithine, Lys – Lysine, Arg – Arginine, Cit – Citrulline, hArg – Homoarginine. Group-wise comparisons via non-parametric testing (p), Holm-Sidak multiple testing correction (corr p). Further characteristics of the animals are given below. BPsys – systolic blood pressure, EF% - left ventricular ejection fraction, E/E’ – measure of diastolic dysfunction.

|  | **G1: obese HFpEF** | | | **G2: obese HFpEF** | | | **G3: lean rats** | | | **G1 vs G3** | | **G2 vs G3** | | **G1 vs G2** | |
| --- | --- | --- | --- | --- | --- | --- | --- | --- | --- | --- | --- | --- | --- | --- | --- |
|  | **mean** | **SD** | **n** | **mean** | **SD** | **n** | **mean** | **SD** | **n** | **p** | **corr p** | **p** | **corr p** | **p** | **corr p** |
| **Arginase 1 ng/ml** | **991** | **519** | **3** | **68.6** | **45.2** | **9** | **17.5** | **11.9** | **12** | **0.000** | **0.000** | **0.001** | **0.029** | **0.000** | **0.004** |
| Orn blood umol/L | 121 | 32 | 3 | 74.2 | 16.3 | 9 | 53.6 | 12.0 | 12 | **0.000** | **0.001** | **0.003** | 0.070 | **0.006** | 0.143 |
| Orn liver | 7.1 | 2.6 | 3 | 6.4 | 1.8 | 9 | 11.5 | 2.3 | 12 | **0.012** | 0.195 | **0.000** | **0.001** | 0.596 | na |
| Orn kidney | 5.8 | 0.0 | 1 | 5.3 | 0.8 | 5 | 4.5 | 1.0 | 6 | 0.294 | na | 0.178 | na | 0.629 | na |
| Orn heart nmol/mg | 1.2 | 0.2 | 3 | 0.000 | 0.000 | 6 | 0.050 | 0.112 | 5 | **0.000** | **0.000** | 0.297 | na | **<1.0E-6** | **0.000** |
| Lys blood umol/L | 347 | 39 | 3 | 444 | 116 | 9 | 581 | 112 | 12 | **0.004** | 0.084 | **0.014** | 0.217 | 0.196 | na |
| Lys liver nmol/mg | 25.0 | 1.1 | 3 | 26.4 | 4.5 | 9 | 31.1 | 6.2 | 12 | 0.116 | na | 0.066 | 0.641 | 0.606 | na |
| Lys kidney nmol/mg | 30.8 | 0.0 | 1 | 33.9 | 5.0 | 5 | 31.6 | 3.0 | 6 | 0.814 | na | 0.378 | na | 0.603 | na |
| Lys heart nmol/mg | 7.5 | 0.3 | 3 | 10.4 | 0.6 | 6 | 16.9 | 3.4 | 5 | **0.004** | 0.075 | **0.001** | **0.027** | **0.000** | **0.004** |
| Arg blood umol/L | 36.7 | 17.1 | 3 | 87.3 | 20.3 | 9 | 109 | 24 | 12 | **0.000** | **0.007** | **0.040** | 0.483 | **0.003** | 0.076 |
| Arg liver nmol/mg | 0.620 | 0.062 | 3 | 0.749 | 0.166 | 9 | 0.648 | 0.118 | 12 | 0.709 | na | 0.118 | na | 0.230 | na |
| Arg kidney nmol/mg | 19.9 | 0.0 | 1 | 19.2 | 2.9 | 5 | 18.0 | 2.7 | 6 | 0.559 | na | 0.515 | na | 0.840 | na |
| Arg heart nmol/mg | 3.3 | 0.4 | 3 | 4.6 | 0.7 | 6 | 6.5 | 1.1 | 5 | **0.003** | 0.065 | **0.006** | 0.115 | **0.014** | 0.264 |
| Cit blood umol/L | 98.9 | 8.4 | 3 | 89.9 | 7.5 | 9 | 77.8 | 10.3 | 12 | **0.006** | 0.115 | **0.008** | 0.140 | 0.109 | na |
| Cit liver nmol/mg | 0.747 | 0.294 | 3 | 0.868 | 0.309 | 9 | 1.0 | 0.3 | 12 | 0.150 | na | 0.249 | na | 0.566 | na |
| Cit kidney nmol/mg | 1.9 | 0.0 | 1 | 1.6 | 0.1 | 5 | 1.5 | 0.2 | 6 | 0.103 | na | 0.223 | na | 0.196 | na |
| Cit heart nmol/mg | 1.7 | 0.2 | 3 | 3.1 | 0.6 | 6 | 3.2 | 0.7 | 5 | **0.012** | 0.195 | 0.872 | na | **0.010** | 0.214 |
| hArg blood umol/L | 0.220 | 0.135 | 3 | 0.651 | 0.358 | 9 | 1.9 | 0.5 | 12 | **0.000** | **0.001** | **<1.0E-5** | **<1.0E-4** | 0.075 | 0.791 |
| hArg liver pmol/mg | 49.0 | 8.6 | 3 | 65.1 | 15.6 | 9 | 101 | 20 | 12 | **0.001** | **0.024** | **<1.0E-3** | **0.008** | 0.127 | na |
| hArg kidney pmol/mg | 62.9 | 0.0 | 1 | 48.0 | 7.8 | 5 | 58.7 | 9.6 | 6 | 0.703 | na | 0.076 | na | 0.156 | na |
| hArg heart pmol/mg | 34.0 | 3.2 | 3 | 31.9 | 8.9 | 6 | 72.1 | 19.9 | 5 | **0.019** | 0.262 | **0.002** | **0.033** | 0.711 | na |
| SDMA blood umol/L | 0.337 | 0.055 | 3 | 0.299 | 0.043 | 9 | 0.301 | 0.028 | 12 | 0.121 | na | 0.901 | na | 0.242 | na |
| SDMA liver pmol/mg | 29.5 | 3.7 | 3 | 29.9 | 6.1 | 9 | 27.3 | 5.3 | 12 | 0.525 | na | 0.318 | na | 0.915 | na |
| SDMA kidney pmol/mg | 52.0 | 0.0 | 1 | 90.3 | 14.8 | 5 | 80.8 | 10.3 | 6 | **0.049** | 0.529 | 0.241 | na | 0.077 | na |
| SDMA heart pmol/mg | 8.4 | 0.0 | 1 | 5.7 | 1.0 | 6 | 9.5 | 1.5 | 5 | 0.533 | na | **0.001** | **0.018** | 0.055 | na |
| ADMA blood umol/L | 0.843 | 0.133 | 3 | 0.841 | 0.121 | 9 | 0.728 | 0.102 | 12 | 0.119 | na | **0.031** | na | 0.979 | na |
| ADMA liver pmol/mg | 41.9 | 4.9 | 3 | 50.0 | 17.6 | 9 | 61.2 | 21.0 | 12 | 0.147 | na | 0.209 | na | 0.465 | na |
| ADMA kidney pmol/mg | 75.7 | 0.0 | 1 | 80.9 | 16.5 | 5 | 90.8 | 22.1 | 6 | 0.555 | na | 0.428 | na | 0.790 | na |
| ADMA heart pmol/mg | 14.7 | 2.4 | 3 | 5.7 | 1.0 | 6 | 9.5 | 1.5 | 5 | **0.008** | 0.134 | **0.001** | **0.018** | **<1.0E-4** | **0.002** |
|  |  |  |  |  |  |  |  |  |  |  |  |  |  |  |  |
| body weight (g) | 494 | 0.693 | 3 | 460 | 22.1 | 9 | 235 | 9.01 | 12 | **<1.0E-6** | nd | **<1.0E-6** | nd | **0.030** | nd |
| tibia (mm) | 36.9 | 0.3 | 3 | 36.9 | 0.567 | 9 | 37.1 | 0.977 | 12 | 0.771 | nd | 0.667 | nd | 0.968 | nd |
| heart weight (mg) | 1350 | 10.0 | 3 | 1390 | 82.0 | 9 | 931 | 50.1 | 12 | **<1.0E-6** | nd | **<1.0E-6** | nd | 0.433 | nd |
| NT-pro-BNP (pg/ml) | 1286 | 310 | 3 | 1171 | 359.6 | 9 | 895 | 371 | 12 | 0.118 | nd | 0.104 | nd | 0.633 | nd |
| BPsys | 174 | 11.5 | 3 | 176 | 16.0 | 8 | 143 | 18.6 | 12 | **0.019** | nd | **0.001** | nd | 0.797 | nd |
| EF % | 83 | 7.8 | 3 | 78.0 | 8.4 | 9 | 78.6 | 9.4 | 9 | 0.481 | nd | 0.897 | nd | 0.388 | nd |
| E/E' | 19.1 | 0.478 | 3 | 22.6 | 3.7 | 9 | 15.0 | 2.8 | 9 | **0.034** | nd | **<1.0E-3** | nd | 0.146 | nd |
| Nitrate blood (µM) | 154.0 | 28.0 | 3 | 134.3 | 42.7 | 9 | 5.3 | 3.7 | 12 | **<1.0E-6** | <1.0E-6 | **<1.0E-6** | <1.0E-6 | 0.478 | na |
| Macrophages in heart | 39 | 32 | 3 | 39 | 12 | 9 | 12 | 8 | 11 | 0.282 | na | **<1.0E-4** | nd | 0.970 | na |
| Nitrate urine (µM) | 121 | 63.46 | 3 | 140 | 29.9 | 9 | 78.8 | 27.7 | 12 | 0.086 | na | **<1.0E-3** | **<1.0E-2** | 0.490 | na |
| Nitrite urine (µM) | 11.2 | 6.0 | 3 | 11.2 | 2.1 | 9 | 5.0 | 2.4 | 12 | **0.010** | 0.128 | **<1.0E-5** | **<1.0E-4** | 0.985 | na |
| Orn+Cit urine (µM) | 8.2 | 5.9 | 3 | 8.5 | 3.3 | 9 | 6.3 | 1.8 | 12 | 0.309 | na | 0.058 | na | 0.903 | na |
| Lys urine (µM) | 25.1 | 27.0 | 3 | 24.5 | 12.0 | 9 | 25.8 | 13.1 | 12 | 0.947 | na | 0.814 | na | 0.955 | na |
| Arg urine (µM) | 6.0 | 5.0 | 3 | 9.9 | 6.0 | 9 | 7.888 | 3.465 | 12 | 0.454 | na | 0.348 | na | 0.342 | na |
| hArg urine (µM) | 0.067 | 0.081 | 3 | 0.072 | 0.034 | 9 | 0.118 | 0.05 | 12 | 0.181 | na | **0.030** | 0.239 | 0.864 | na |
| ADMA urine (µM) | 0.38 | 0.498 | 3 | 0.232 | 0.144 | 9 | 0.198 | 0.085 | 12 | 0.202 | na | 0.497 | na | 0.409 | na |
| DMA urine (µM) | 61.4 | 40.7 | 3 | 73.9 | 20.5 | 9 | 78.3 | 27.2 | 12 | 0.392 | na | 0.692 | na | 0.482 | na |


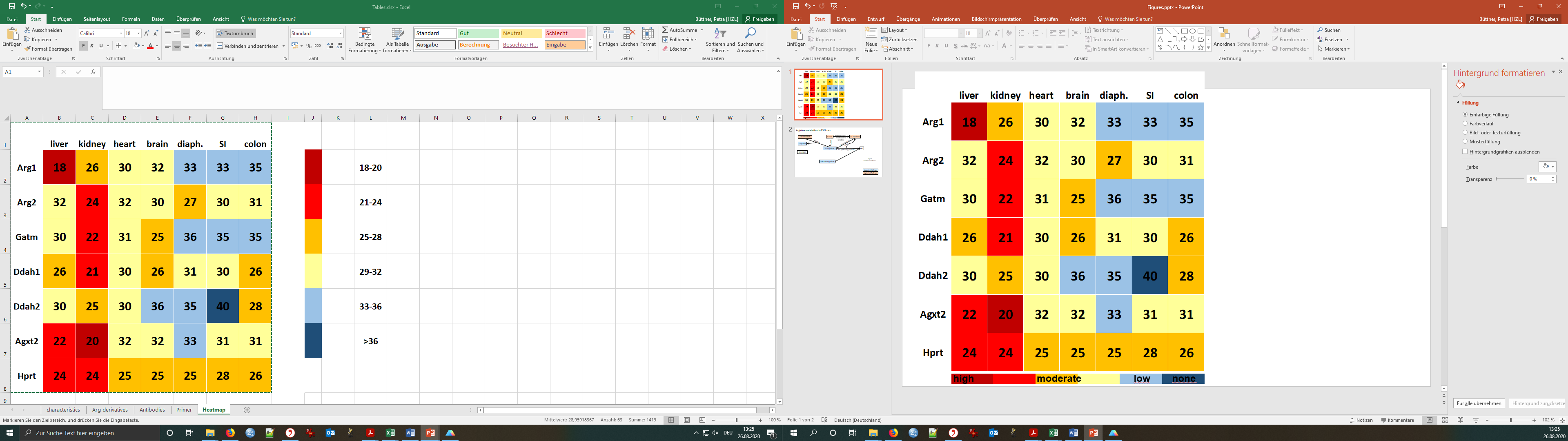


**Supplementary figure 1:** Heatmap of the gene expression of key enzymes in arginine metabolism in different rat organs. Hprt (Hypoxanthine Phosphoribosyltransferase 1) served as housekeeping gene and is shown at the bottom of this figure for comparison. Numbers indicate the cycle of earliest detection in either lean or obese ZSF1 rats in quantitative Realtime-PCR. Colors indicate the expression range. Dark red = high expression. Dark blue = no expression. diaph. = diaphragm. SI = small intestine.


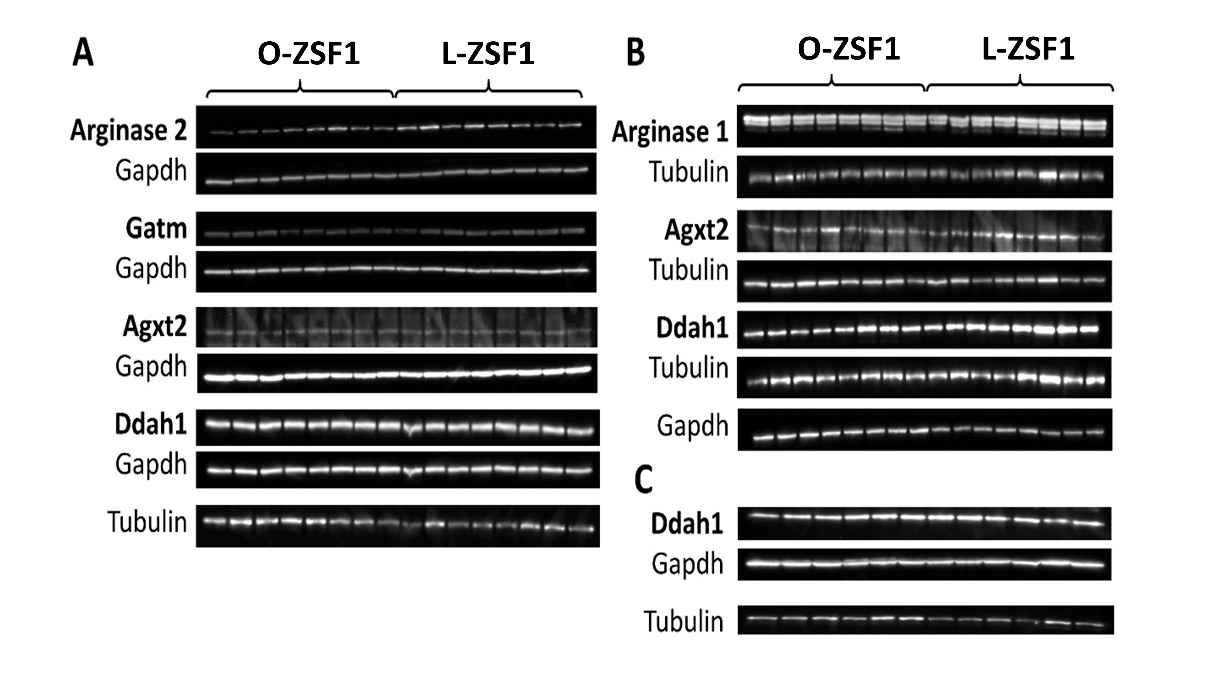


**Supplementary Figure 2:** Summary of Western Blot analysis of key enzymes in arginine metabolism in kidney (A), liver (B) and heart (C). Each protein is shown with the according normalization blot detecting Gapdh or alpha Tubulin. Below each organ panel one example for regulated Gapdh (liver) or alpha Tubulin (kidney/ heart) is shown. All blots in this figure are shown as uncropped as single files below (see figure 6-).


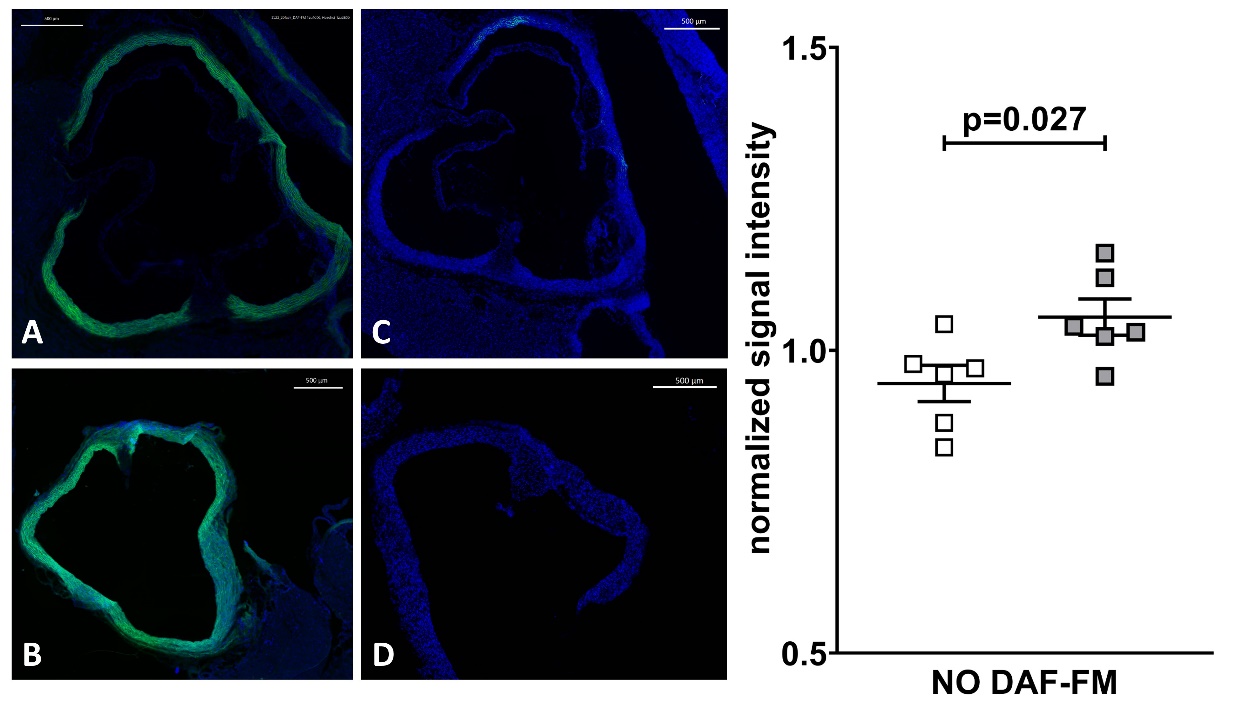


**Supplementary Figure 3:** Left side - Exemplarily visualization of in vitro NO production in aortic root cryosections from a L-ZSF1 rat (panel A/C) and an O-ZSF1 (panel B/C). Cell nuclei were visualized by HOECHST staining (blue). Sections were incubated with DAF-FM, which reacts with NO to fluorescent Benzotriazole detectable at 515nm (green, panel A/B). Preincubation with the NOS antagonist L-NAME almost completely diminished the DAF-FM signal (panel C/D). Right side – Fluorescence signal in O-ZSF1 (grey box) normalized to the signal in L-ZSF1 (white box). Boxplots visualize the median, 25^th^ and 75^th^ percentiles and minimum/ maximum.


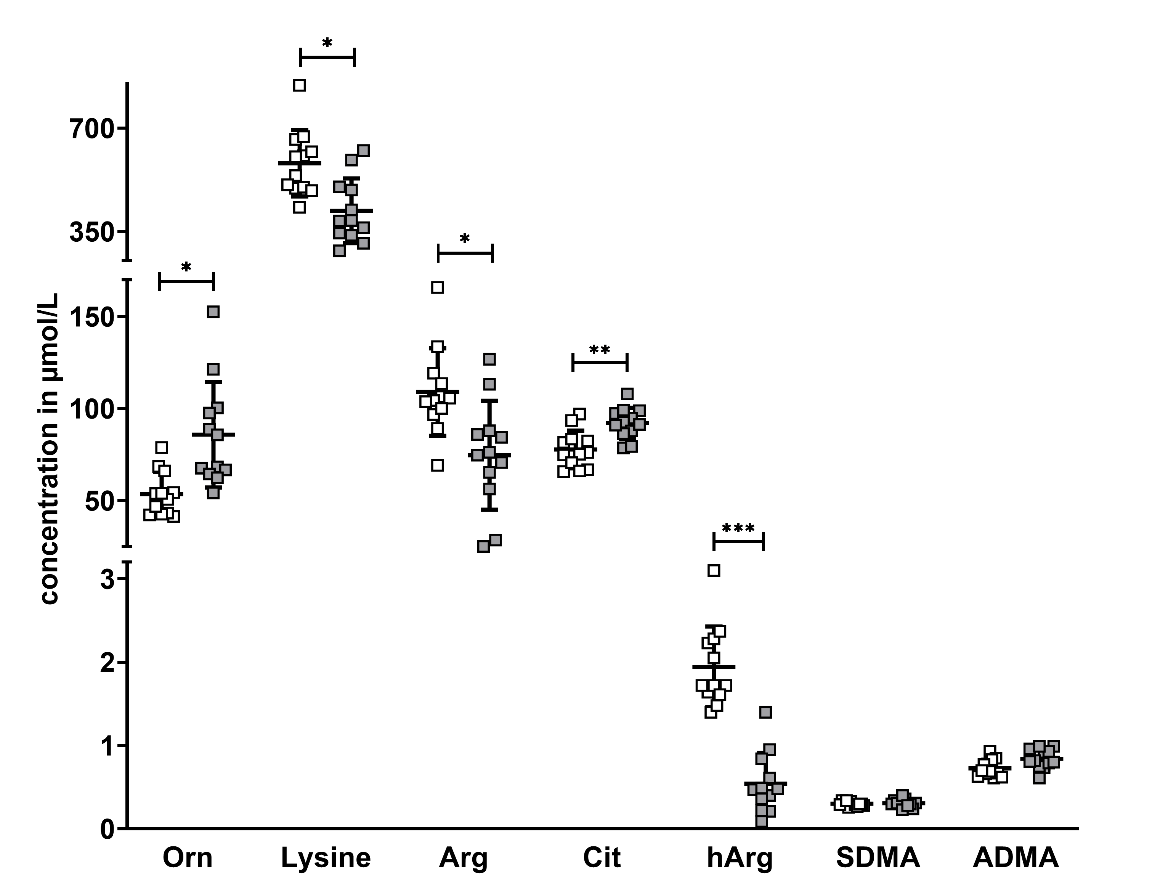


**Supplementary Figure 4:** Boxplots visualizing the median, 25^th^ and 75^th^ percentiles and minimum/ maximum concentrations (µmol/L) of Arg and Arg-related amino acids in L-ZSF1 (white squares) and O-ZSF1 (grey squares). P-values were calculated using Kruskal-Wallis test and corrected for multiple testing using the Bonferroni-Sidak method. * p<0.05, ** p<0.01, *** p<0.001


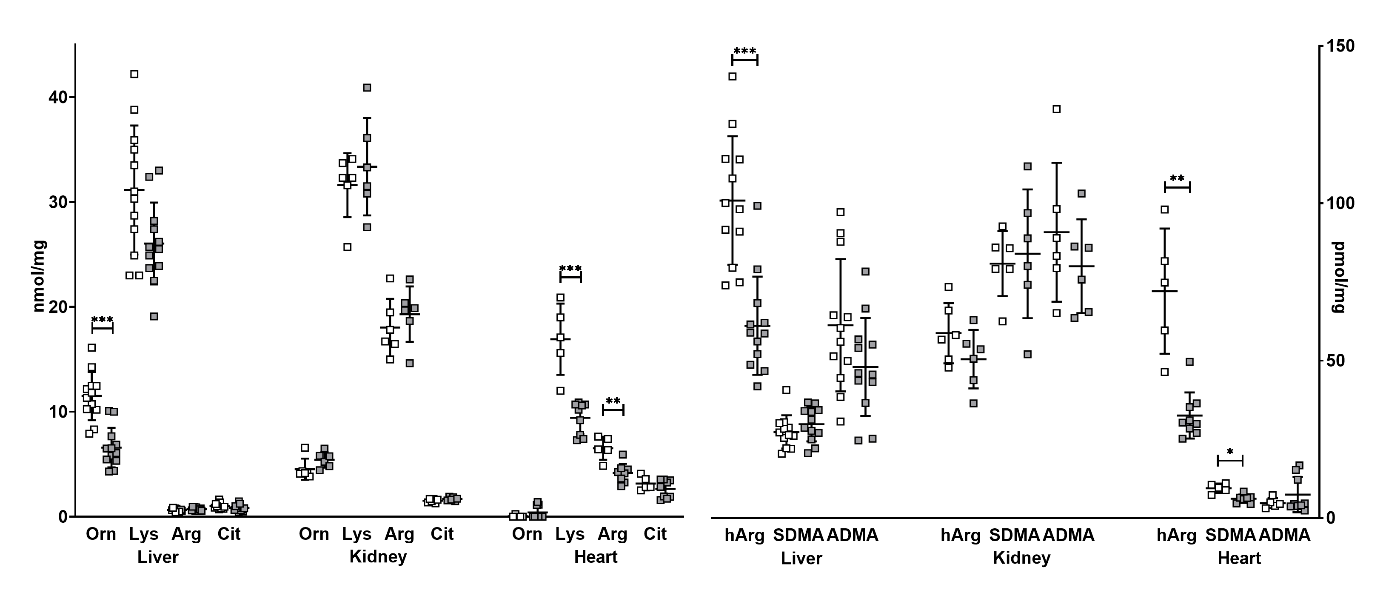


**Supplementary Figure 5:** Tissue content of Arg and Arg-related amino acids in liver, kidney and heart of L-ZSF1 (white squares) and O-ZSF1 (grey squares). Concentrations were normalized to the total protein content of the sample. Boxplots visualize the median, 25^th^ and 75^th^ percentiles and minimum/ maximum. P-values were calculated using Kruskal-Wallis test and corrected for multiple testing using the Bonferroni-Sidak method. * p<0.05, ** p<0.01, *** p<0.001

**Supplementary Materials and Methods**

*Determination of arginine derivatives in serum*

A stable isotope dilution assay for the liquid chromatography-tandem mass spectroscopy (LC-MS/MS) determination of arginine, hArg, ADMA and SDMA was applied which was developed, validated and published previously by the author’s lab [1, 2]. For protein precipitation, a 96-well 0.2 µm microfiltration plate was place on top of a 96-well u-shaped polypropylene autosampler plate. Each well of the upper plate was filled with 2H7-arginine, 13C6-homoarginine, and 2H6-ADMA solved in 100 µL methanol and 25 µL EDTA plasma sample, calibrator, or quality control sample were added. Stable isotope labeled internal standard concentrations corresponding to concentrations of 40, 10 and 2 µM for the analytes arginine, hArg, ADMA and SDMA, respectively. 2H6-ADMA was used as internal standard for ADMA and SDMA. After centrifugation for protein precipitation by filtration and subsequent evaporation of the eluent to dryness by heating to 65 °C, the analytes were converted into their butyl esters derivatives with butanolic HCL at 85 °C. Analyte concentrations were calculated using calibration curves based on four levels in triplicates. The analytes were added to dialyzed plasma previously obtained by dialyzing 20 mL pooled human EDTA plasma with a Slid-A-Lyzer™ cassette against 1,5 L saline three times for 24 hours. The following calibration levels were used for the analytes. Arginine 0, 60, 120, and 250 µM. hArg 0, 2, 5, and 10 µM. ADMA 0, 0.5, 1, and 2 µM. SDMA 0, 0.5, 1, and 2 µM. Calibration curves for each analyte were generated by plotting the calibration level (x-axis) against the peak area ratios of the analyte divided by the internal standard (y-axis). The analyte concentration in a plasma EDTA or quality control sample was calculated by the corresponding peak area ratios divided by the slope of the calibration curve.

1. Atzler, D.; Mieth, M.; Maas, R.; Böger, R.H.; Schwedhelm, E. Stable isotope dilution assay for liquid chromatography-tandem mass spectrometric determination of L-homoarginine in human plasma. J. Chromatogr. B Analyt. Technol. Biomed. Life Sci. 2011, 879, 2294–2298, doi:10.1016/j.jchromb.2011.06.016.

2. Schwedhelm, E.; Maas, R.; Tan-Andresen, J.; Schulze, F.; Riederer, U.; Böger, R.H. High-throughput liquid chromatographic-tandem mass spectrometric determination of arginine and dimethylated arginine derivatives in human and mouse plasma. J. Chromatogr. B Analyt. Technol. Biomed. Life Sci. 2007, 851, 211–219, doi:10.1016/j.jchromb.2006.11.052.

**Supplementary Figure 6:** Original, unprocessed full length blot of Arginase 2 (top) in kidney lysates of eight O-ZSF1 (left) and eight L-ZSF1 rats. Glyceraldehyde-3-phosphate dehydrogenase was used for concentration normalization (bottom). The left pictures are overlays of bright field and chemiluminescence to visualize the protein ladder (PageRuler Prestained Protein ladder Plus, ThermoFisher, Waltham, USA) together with the bands. On the right side, the according chemiluminescence signal of the blot that was used for signal calculation is shown. The analyzed band is indicated with an arrow. The Protein ladder was adapted to the specific blot in every picture.


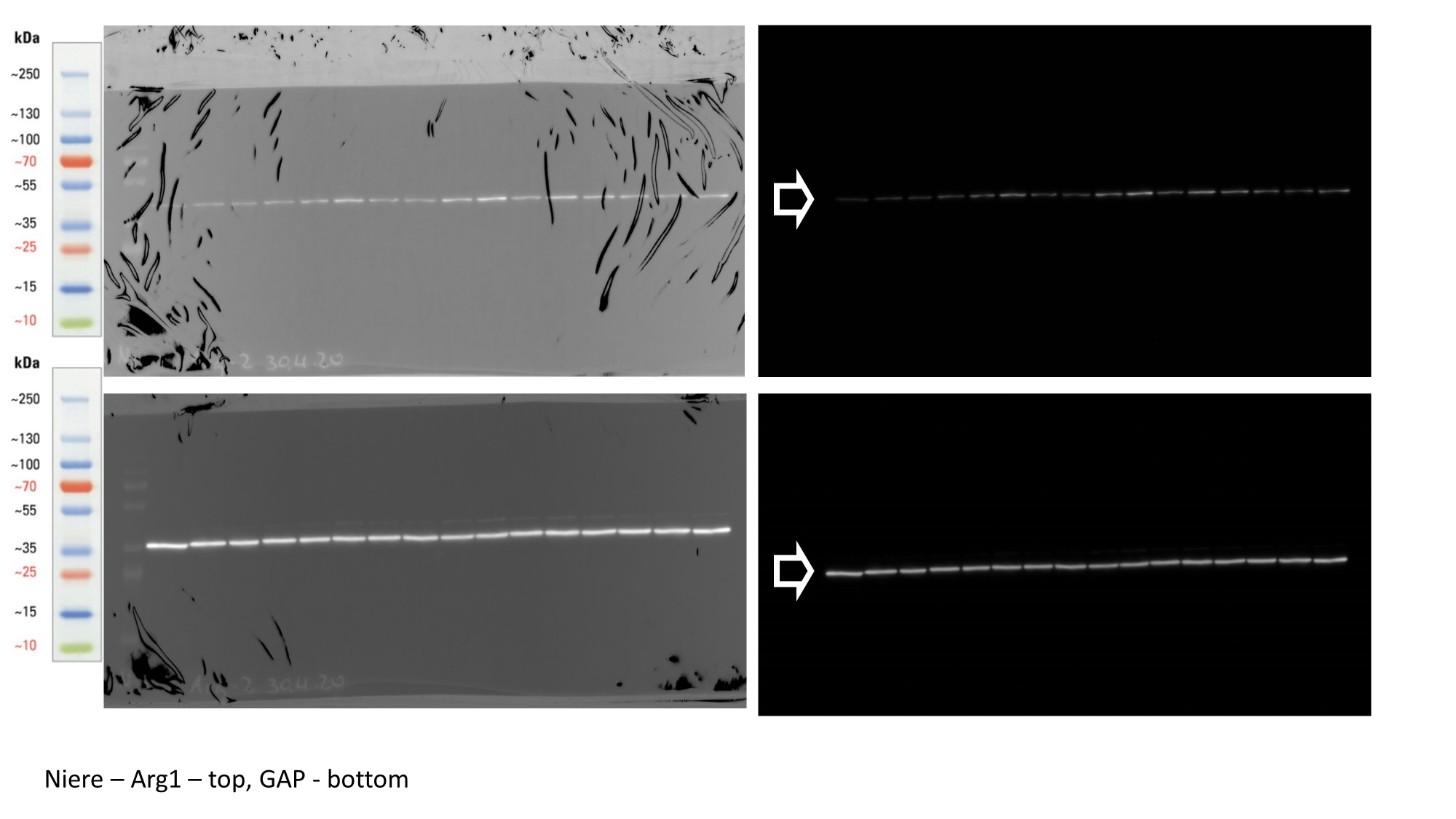


**Supplementary Figure 7:** Original, unprocessed full length blot of Glycine Amidinotransferase (top) in kidney lysates of eight O-ZSF1 (left) and eight L-ZSF1 rats. Glyceraldehyde-3-phosphate dehydrogenase was used for concentration normalization (bottom). The left pictures are overlays of bright field and chemiluminescence to visualize the protein ladder (PageRuler Prestained Protein ladder Plus, ThermoFisher, Waltham, USA) together with the bands. On the right side, the according chemiluminescence signal of the blot that was used for signal calculation is shown. The analyzed band is indicated with an arrow. The Protein ladder was adapted to the specific blot in every picture.


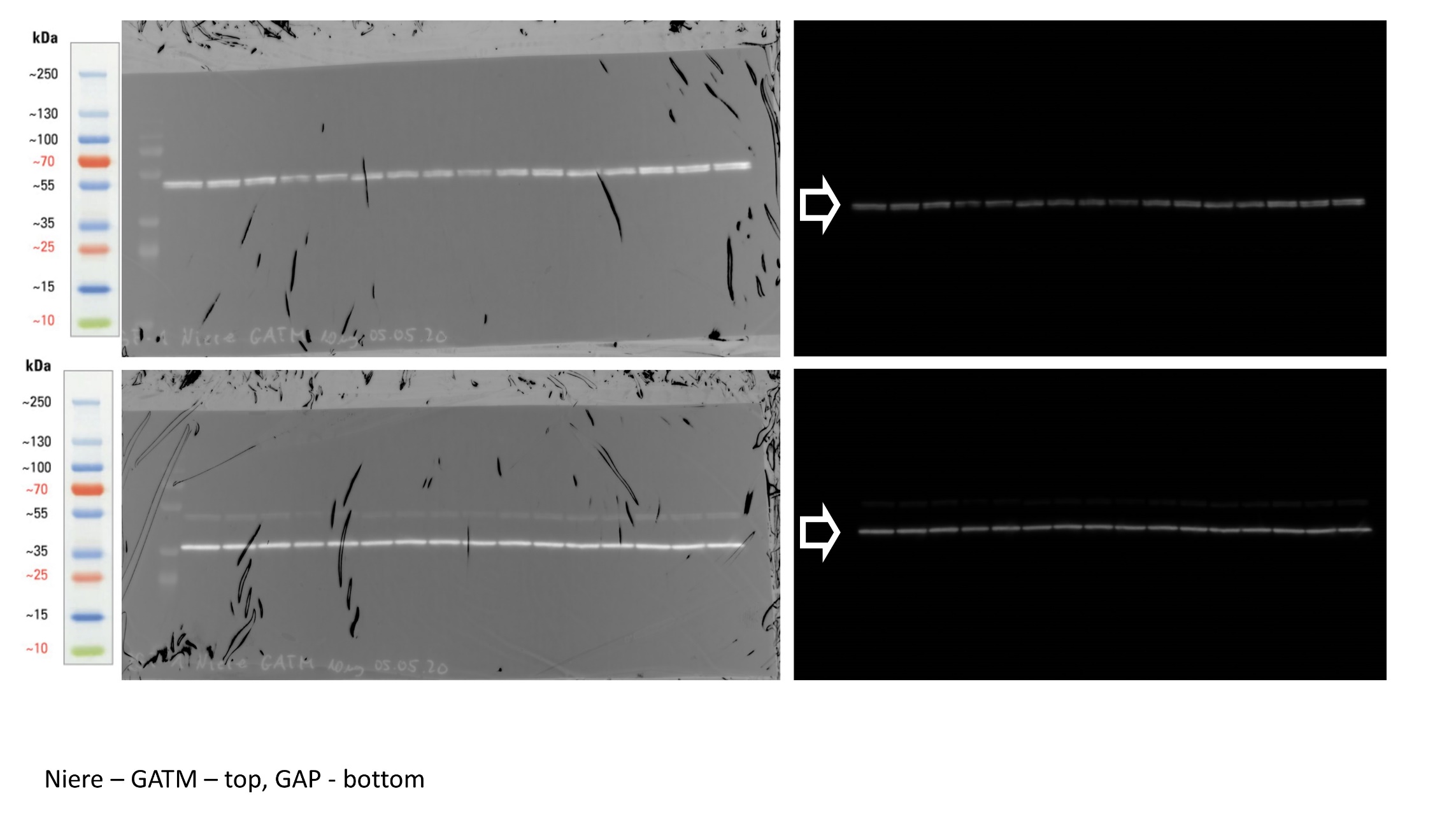


**Supplementary Figure 8:** Original, unprocessed full length blot of Alanine-Glyoxylate Aminotransferase 2 (top) in kidney lysates of eight O-ZSF1 (left) and eight L-ZSF1 rats. Glyceraldehyde-3-phosphate dehydrogenase was used for concentration normalization (bottom). The left pictures are overlays of bright field and chemiluminescence to visualize the protein ladder (PageRuler Prestained Protein ladder Plus, ThermoFisher, Waltham, USA) together with the bands. On the right side, the according chemiluminescence signal of the blot that was used for signal calculation is shown. The analyzed band is indicated with an arrow. The Protein ladder was adapted to the specific blot in every picture.


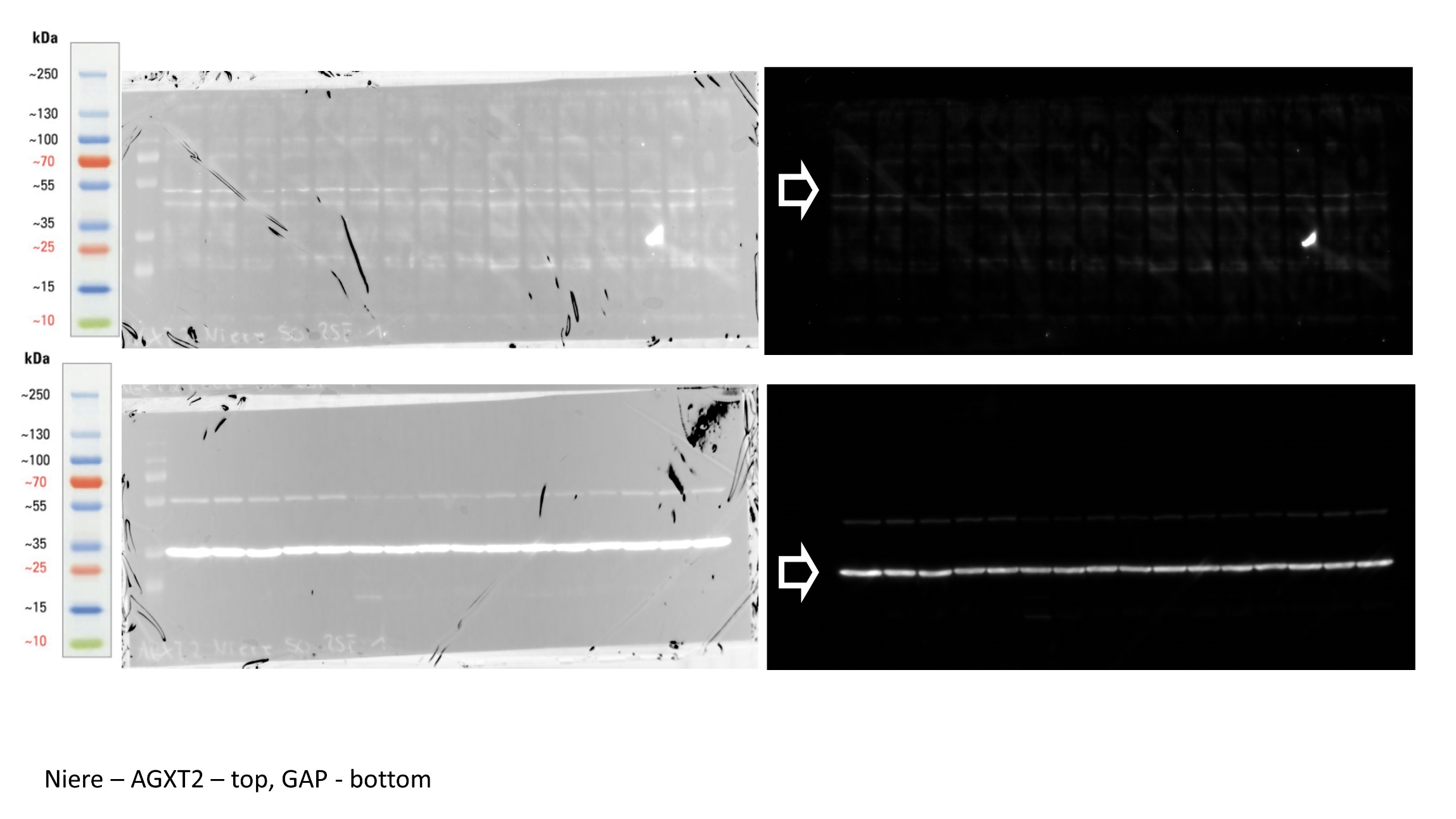


**Supplementary Figure 9:** Original, unprocessed full length blot of Dimethylarginine Dimethylaminohydrolase 1 (top) in kidney lysates of eight O-ZSF1 (left) and eight L-ZSF1 rats. Glyceraldehyde-3-phosphate dehydrogenase was used for concentration normalization (bottom). The left pictures are overlays of bright field and chemiluminescence to visualize the protein ladder (PageRuler Prestained Protein ladder Plus, ThermoFisher, Waltham, USA) together with the bands. On the right side, the according chemiluminescence signal of the blot that was used for signal calculation is shown. The analyzed band is indicated with an arrow. The Protein ladder was adapted to the specific blot in every picture.


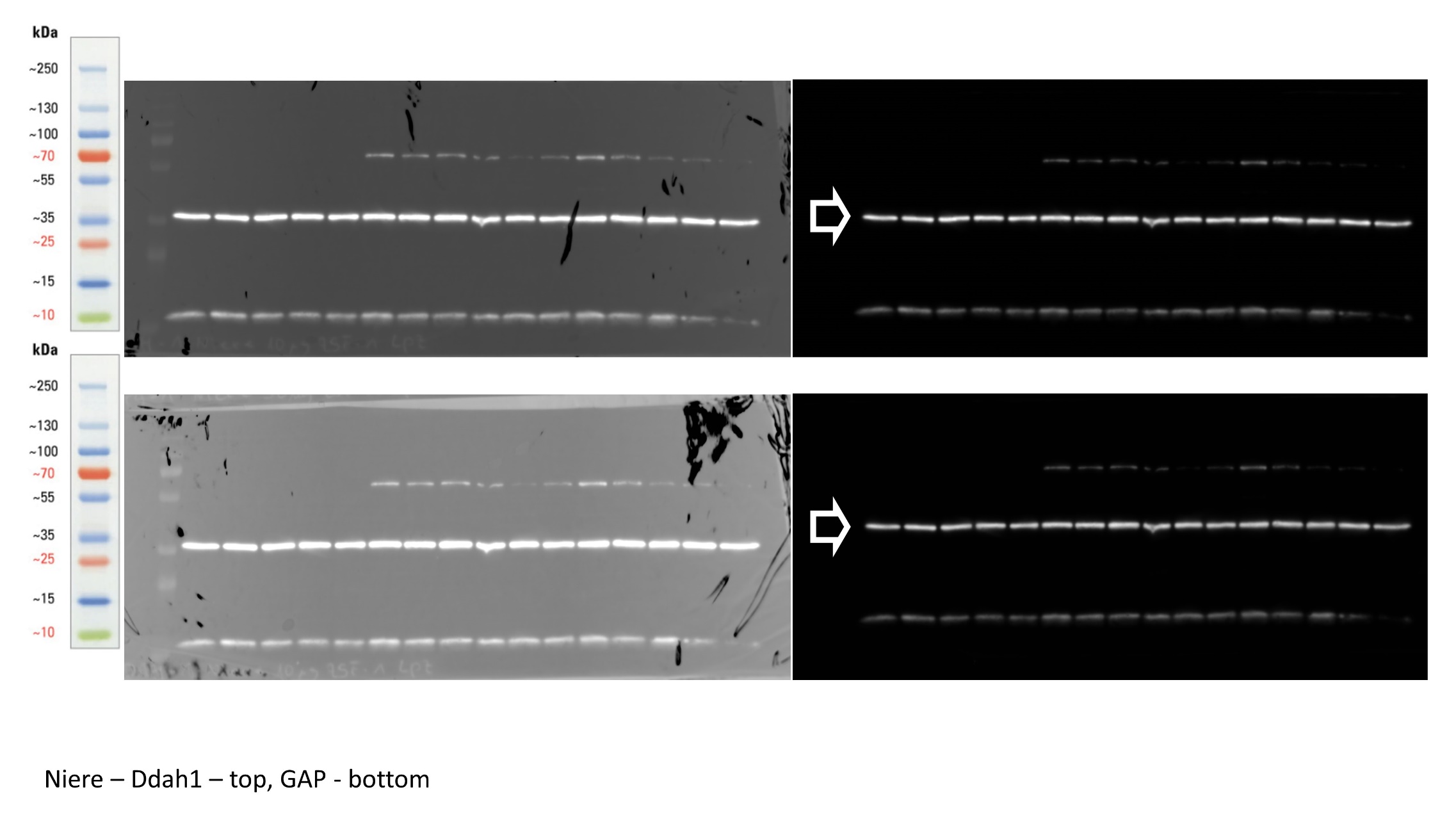


**Supplementary Figure 10:** Original, unprocessed full length blot of alpha tubulin in kidney lysates of eight O-ZSF1 (left) and eight L-ZSF1 rats. The left picture is an overlay of bright field and chemiluminescence to visualize the protein ladder (PageRuler Prestained Protein ladder Plus, ThermoFisher, Waltham, USA) together with the bands. On the right side, the according chemiluminescence signal of the blot that was used for signal calculation is shown. The analyzed band is indicated with an arrow. The Protein ladder was adapted to the specific blot in every picture.


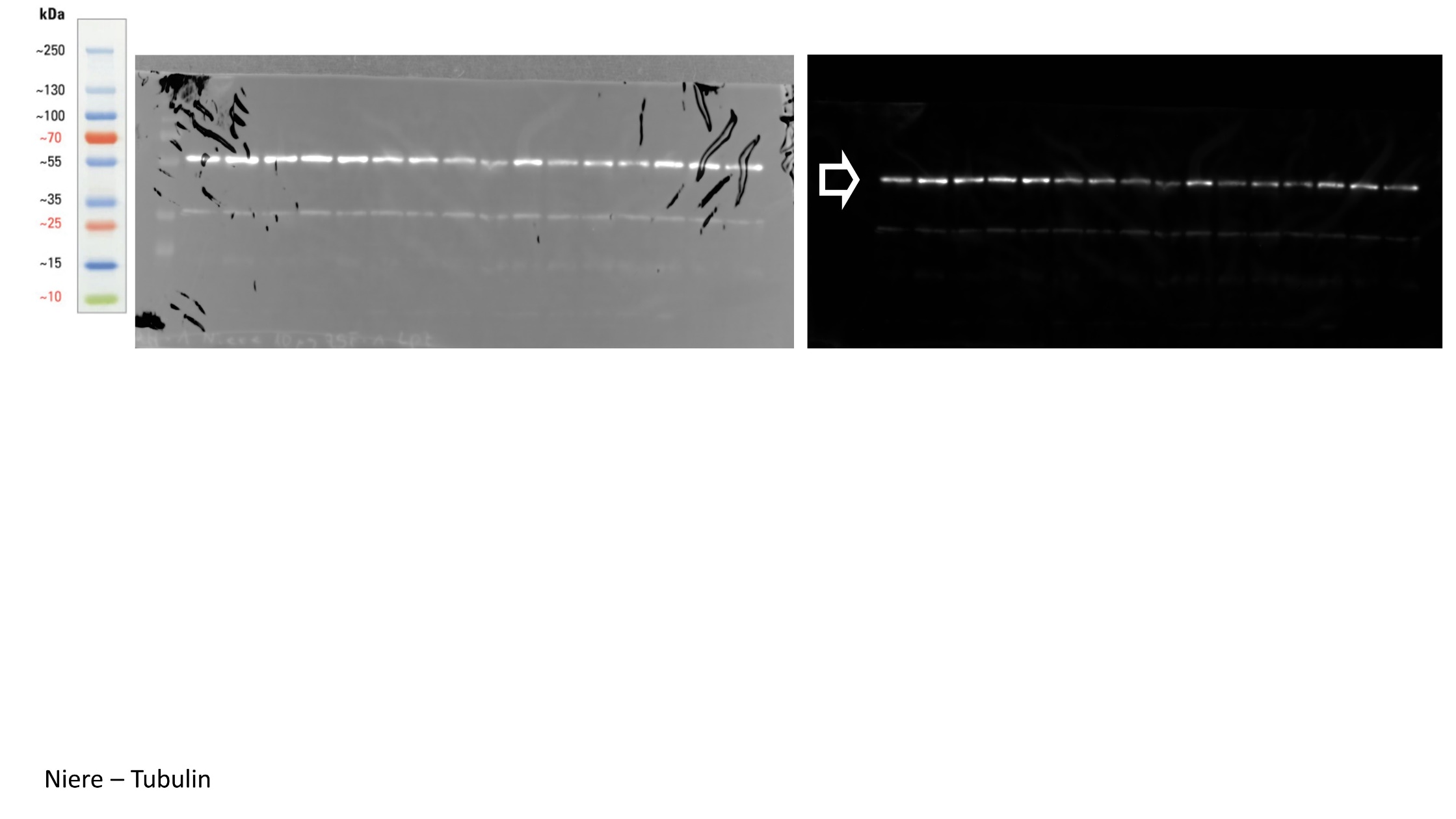


**Supplementary Figure 11:** Original, unprocessed full length blot of Arginase 1 (top) in liver lysates of eight O-ZSF1 (left) and eight L-ZSF1 rats. Alpha tubulin was used for concentration normalization (bottom). The left pictures are overlays of bright field and chemiluminescence to visualize the protein ladder (PageRuler Prestained Protein ladder Plus, ThermoFisher, Waltham, USA) together with the bands. On the right side, the according chemiluminescence signal of the blot that was used for signal calculation is shown. The analyzed band is indicated with an arrow. The Protein ladder was adapted to the specific blot in every picture.


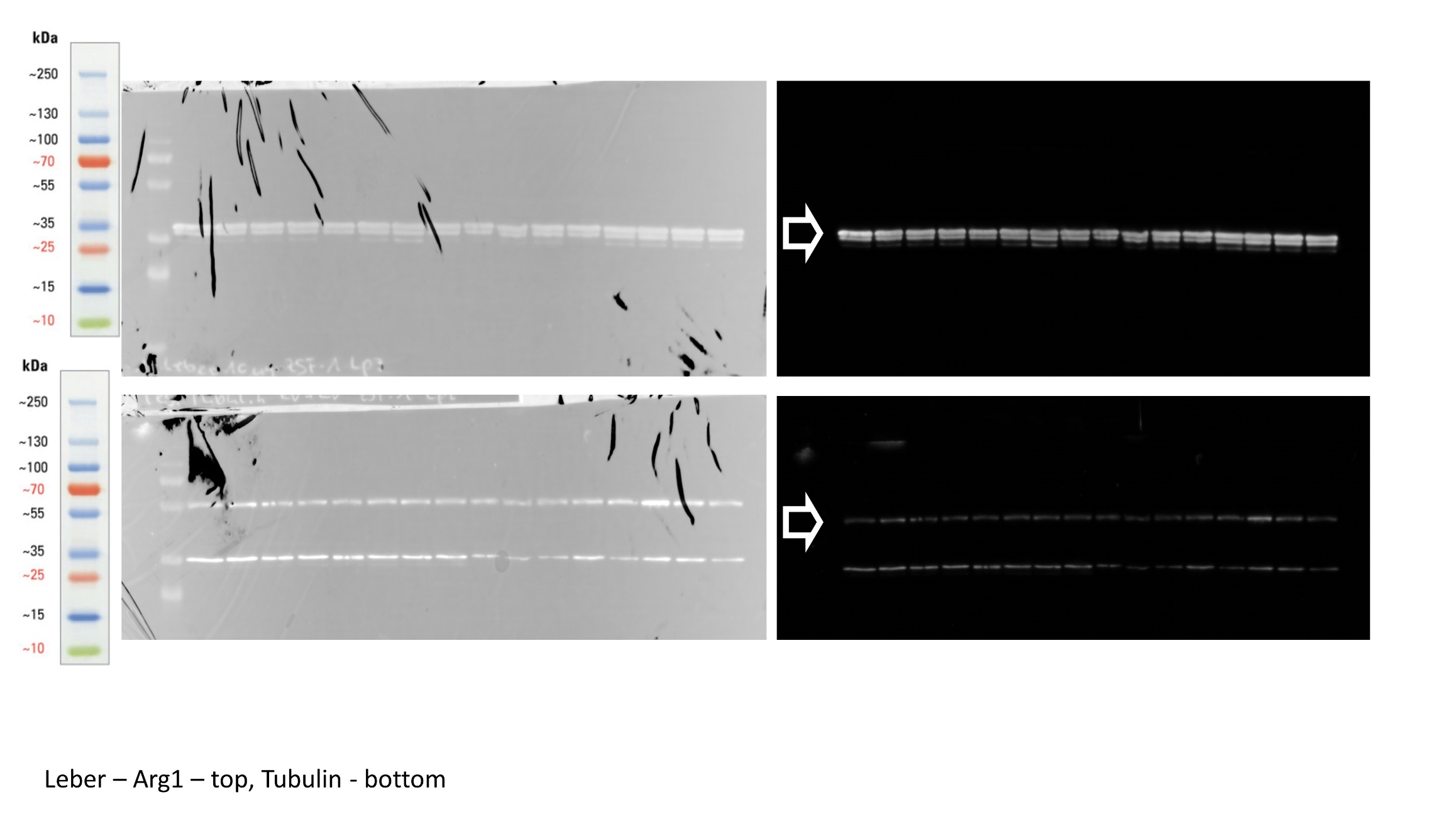


**Supplementary Figure 12:** Original, unprocessed full length blot of Alanine-Glyoxylate Aminotransferase 2 (top) in liver lysates of eight O-ZSF1 (left) and eight L-ZSF1 rats. Alpha tubulin was used for concentration normalization (bottom). The left pictures are overlays of bright field and chemiluminescence to visualize the protein ladder (PageRuler Prestained Protein ladder Plus, ThermoFisher, Waltham, USA) together with the bands. On the right side, the according chemiluminescence signal of the blot that was used for signal calculation is shown. The analyzed band is indicated with an arrow. The Protein ladder was adapted to the specific blot in every picture.


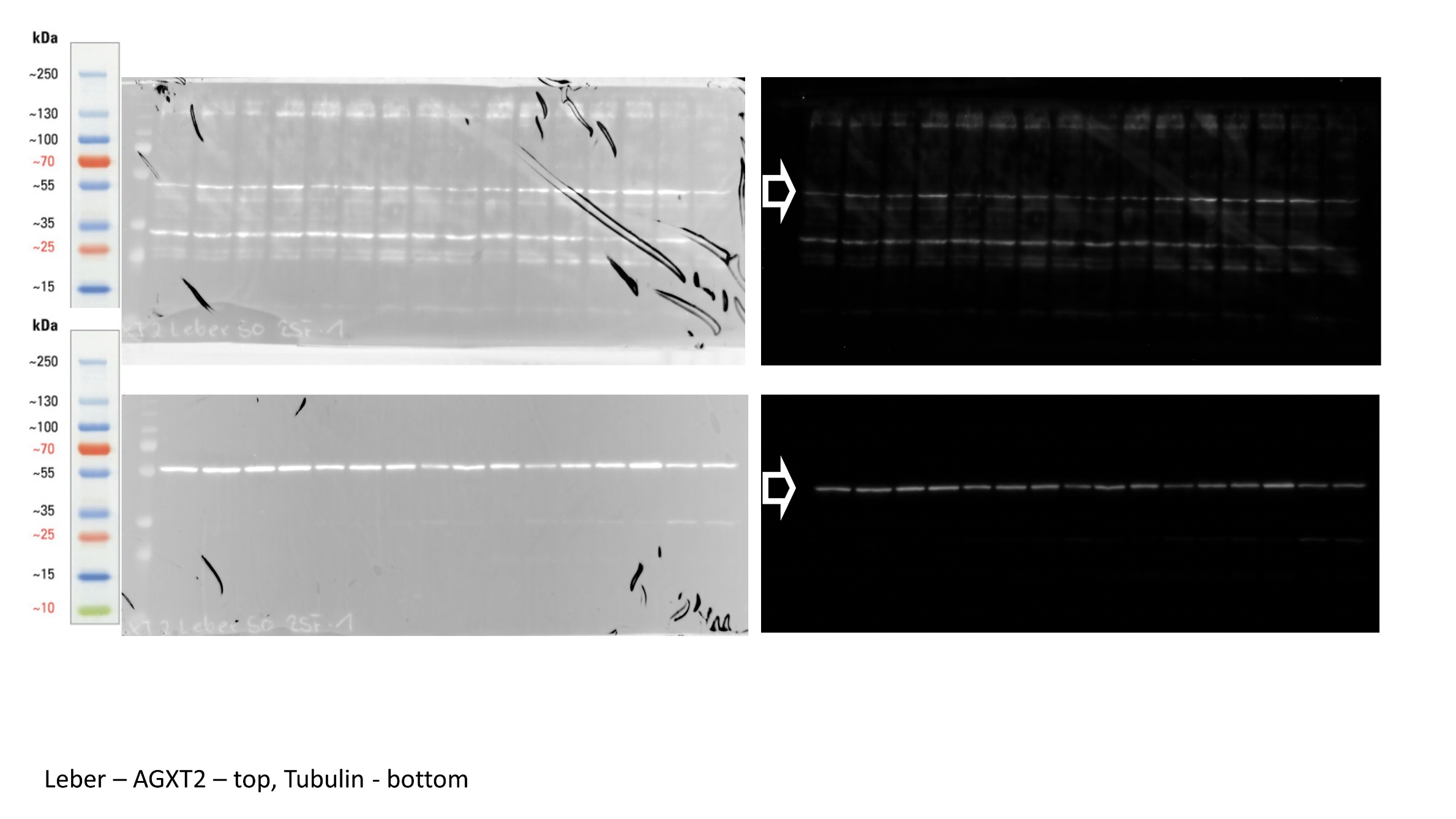


**Supplementary Figure 13:** Original, unprocessed full length blot of Dimethylarginine Dimethylaminohydrolase 1 (top) in liver lysates of eight O-ZSF1 (left) and eight L-ZSF1 rats. Alpha tubulin was used for concentration normalization (bottom). The left pictures are overlays of bright field and chemiluminescence to visualize the protein ladder (PageRuler Prestained Protein ladder Plus, ThermoFisher, Waltham, USA) together with the bands. On the right side, the according chemiluminescence signal of the blot that was used for signal calculation is shown. The analyzed band is indicated with an arrow. The Protein ladder was adapted to the specific blot in every picture.


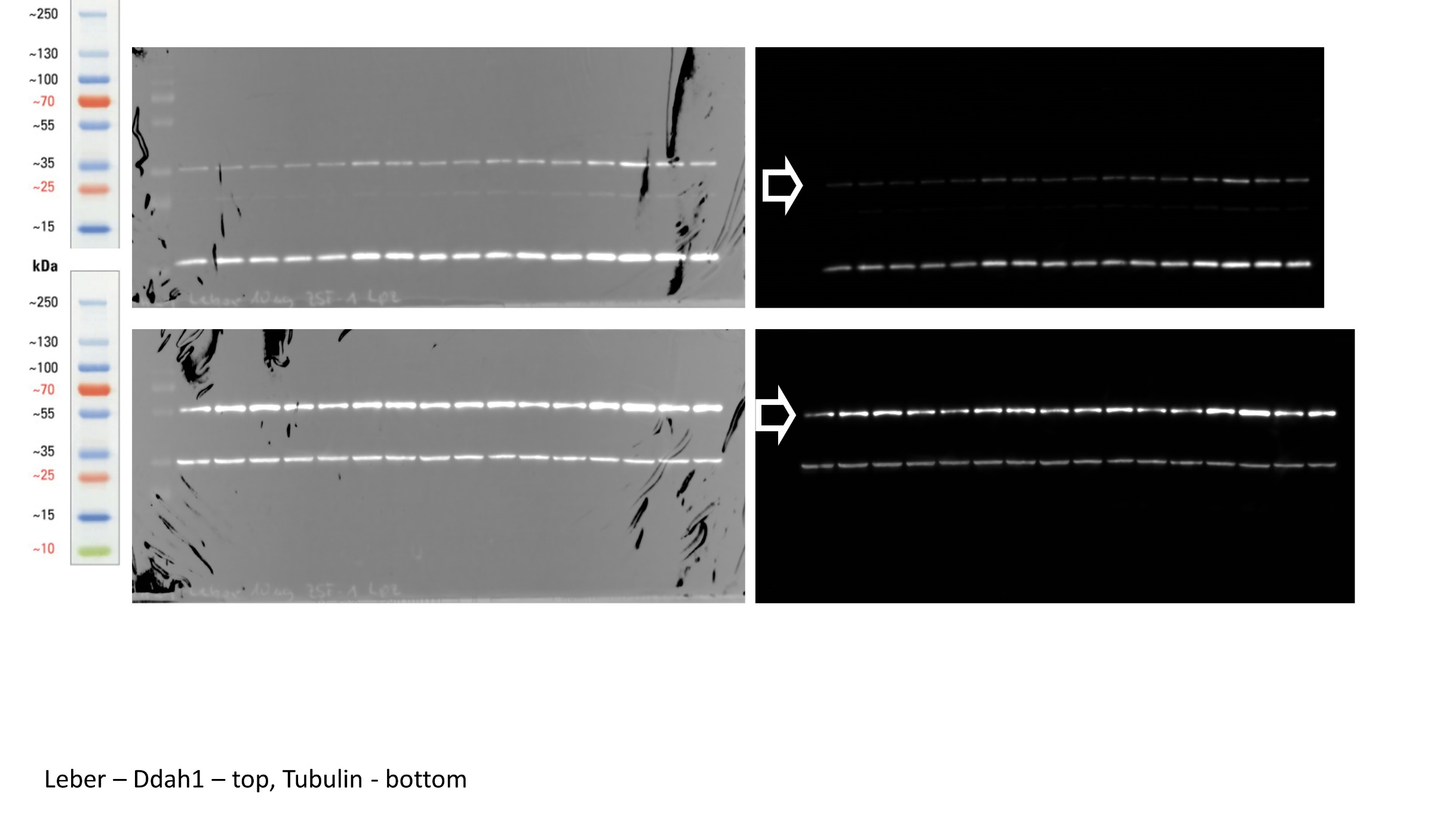


**Supplementary Figure 14:** Original, unprocessed full length blot of Glyceraldehyde-3-phosphate dehydrogenase in liver lysates of eight O-ZSF1 (left) and eight L-ZSF1 rats. The left picture is an overlay of bright field and chemiluminescence to visualize the protein ladder (PageRuler Prestained Protein ladder Plus, ThermoFisher, Waltham, USA) together with the bands. On the right side, the according chemiluminescence signal of the blot that was used for signal calculation is shown. The analyzed band is indicated with an arrow. The Protein ladder was adapted to the specific blot in every picture.


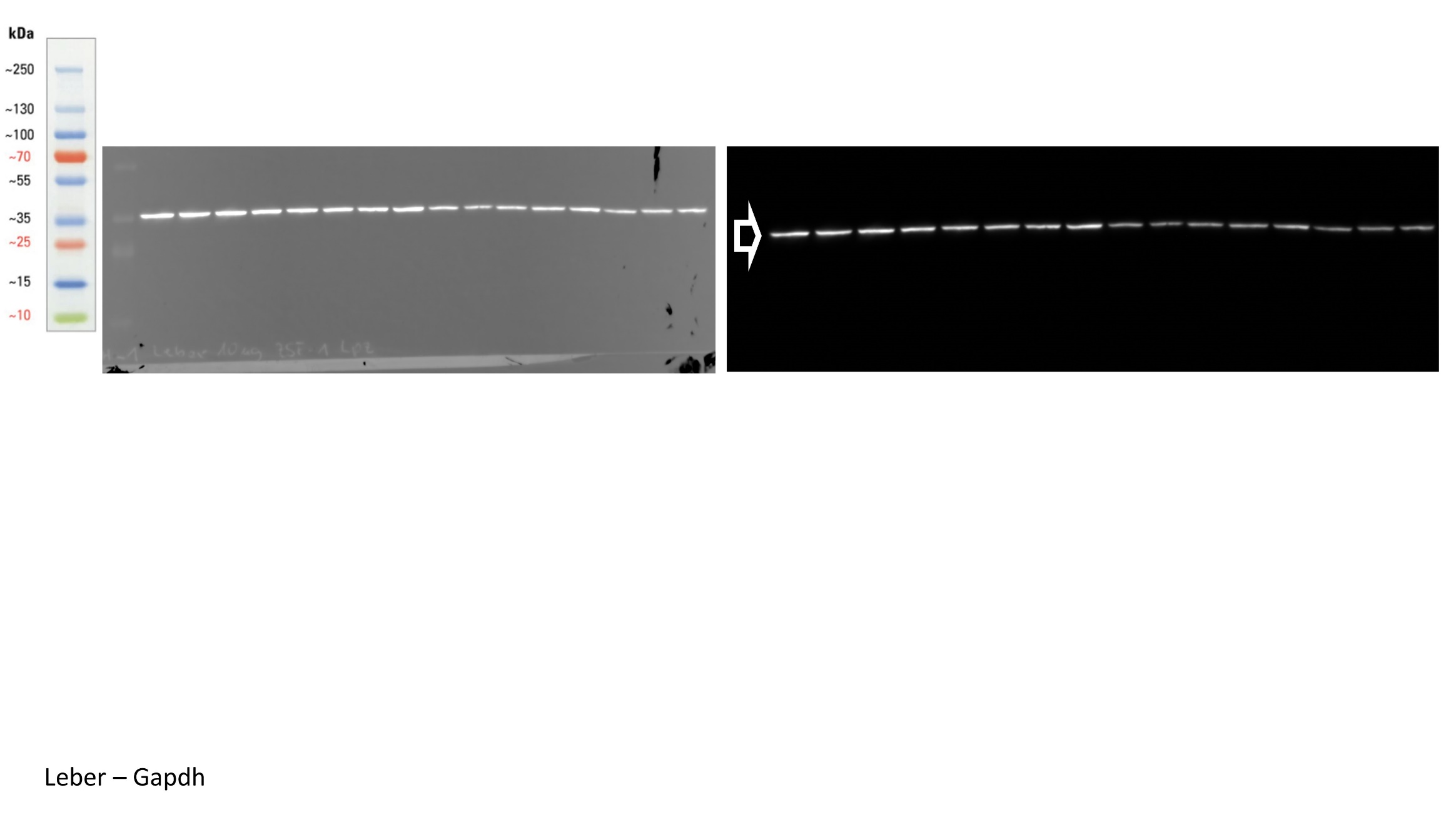


**Supplementary Figure 15:** Original, unprocessed full length blot of Dimethylarginine Dimethylaminohydrolase 1 (top) in left ventricle lysates of six O-ZSF1 (left) and six L-ZSF1 rats. Glyceraldehyde-3-phosphate dehydrogenase was used for concentration normalization (bottom). The left pictures are overlays of bright field and chemiluminescence to visualize the protein ladder (PageRuler Prestained Protein ladder Plus, ThermoFisher, Waltham, USA) together with the bands. On the right side, the according chemiluminescence signal of the blot that was used for signal calculation is shown. The analyzed band is indicated with an arrow. The Protein ladder was adapted to the specific blot in every picture.


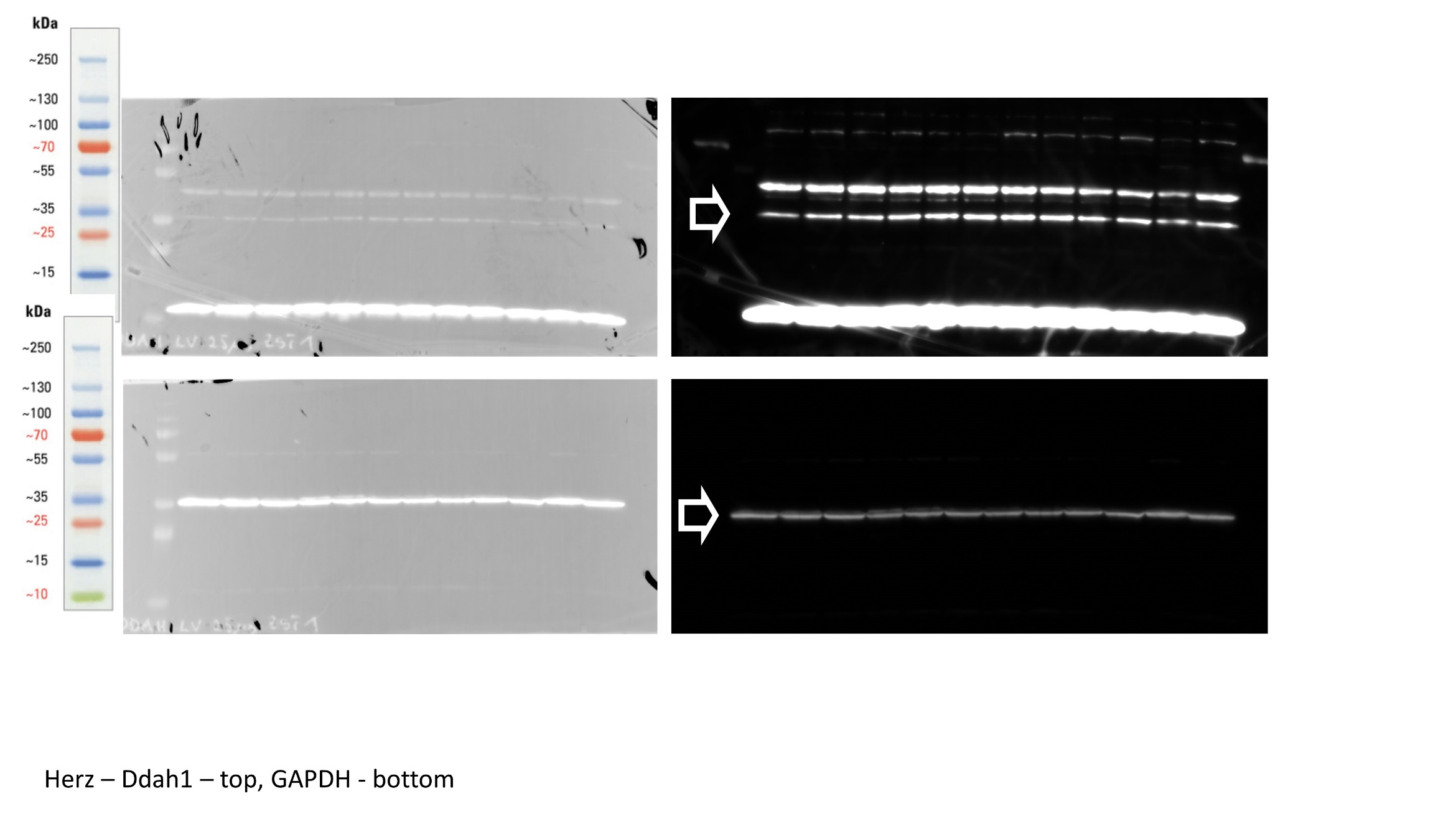


**Supplementary Figure 16:** Original, unprocessed full length blot of alpha Tubulin in left ventricle lysates of six O-ZSF1 (left) and six L-ZSF1 rats. The left picture is an overlay of bright field and chemiluminescence to visualize the protein ladder (PageRuler Prestained Protein ladder Plus, ThermoFisher, Waltham, USA) together with the bands. On the right side, the according chemiluminescence signal of the blot that was used for signal calculation is shown. The analyzed band is indicated with an arrow. The Protein ladder was adapted to the specific blot in every picture. In this example alpha tubulin detection was done following Ddah1 detection.


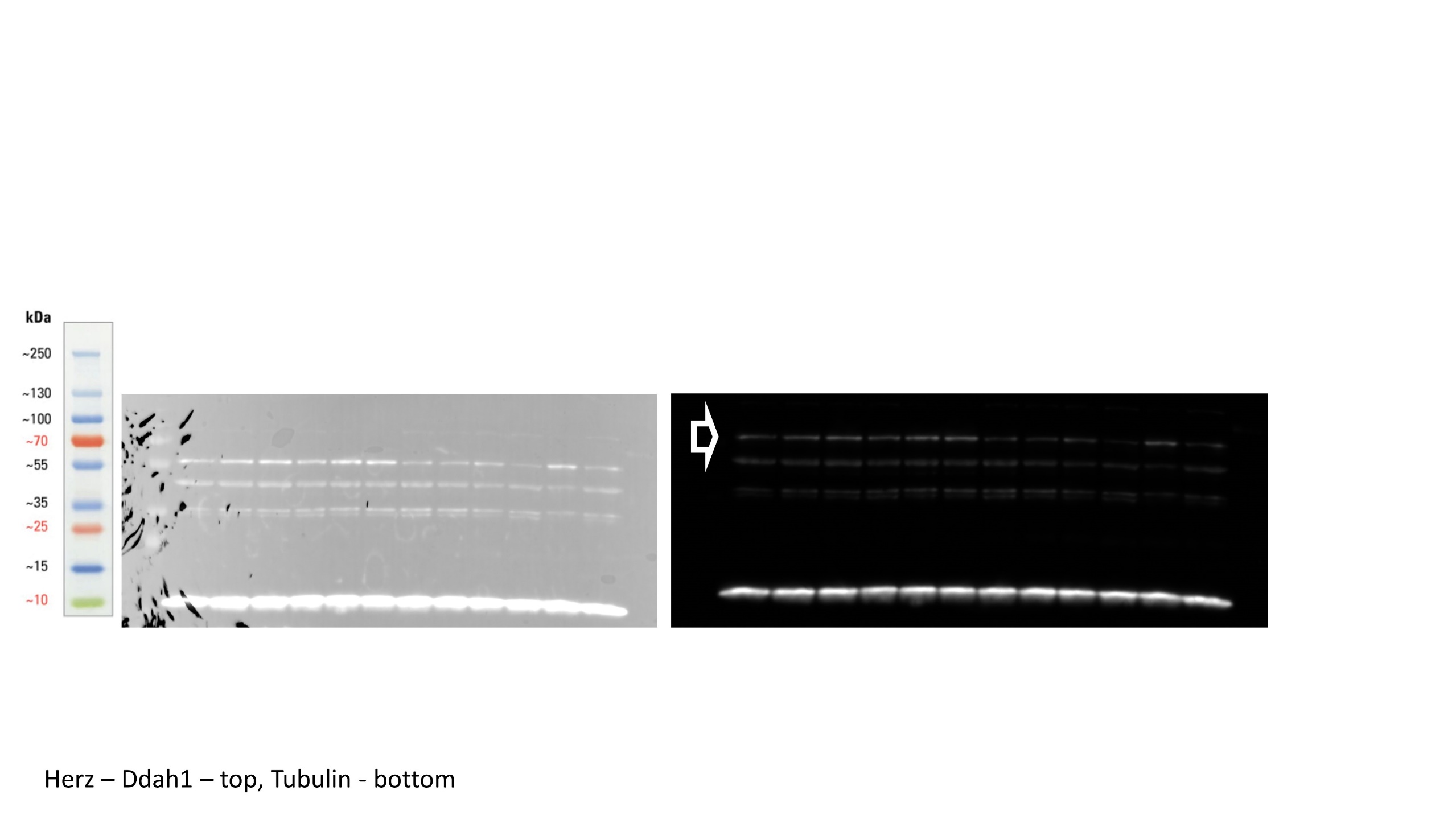

Supplement: Supplementary file 1 — Supplementary Information. [file 41598_2021_216_MOESM1_ESM.docx]
